# Indole-Terpenoids With Anti-inflammatory Activities From *Penicillium* sp. HFF16 Associated With the Rhizosphere Soil of *Cynanchum bungei* Decne

**DOI:** 10.3389/fmicb.2021.710364

**Published:** 2021-07-09

**Authors:** Guojun Pan, Yanfen Zhao, Shuang Ren, Fengyang Liu, Qicai Xu, Weibin Pan, Tongtao Yang, Mingtian Yang, Xinru Zhang, Chuanyue Peng, Gangping Hao, Fandong Kong, Liman Zhou, Na Xiao

**Affiliations:** ^1^College of Life Sciences, Shandong First Medical University & Shandong Academy of Medical Sciences, Tai’an, China; ^2^Key Laboratory of Chemistry and Engineering of Forest Products, State Ethnic Affairs Commission, Guangxi Key Laboratory of Chemistry and Engineering of Forest Products, Guangxi Collaborative Innovation Center for Chemistry and Engineering of Forest Products, School of Chemistry and Chemical Engineering, Guangxi University for Nationalities, Nanning, China; ^3^State Key Laboratory of Crop Biology, College of Agronomy, Shandong Agriculture University, Tai’an, China; ^4^State Key Laboratory of Natural Medicines, China Pharmaceutical University, Nanjing, China

**Keywords:** fungus, *Penicillium* sp. HFF16, indole-terpenoids, anti-inflammatory activity, *Cynanchum bungei* Decne

## Abstract

Four new indole-terpenoids **(1–4)** named encindolene A, 18-*O*-methyl-encindolene A, encindolene B, and encindolene C, as well as three known analogs **(5–7)**, were isolated from the fungus *Penicillium* sp. HFF16 from the rhizosphere soil of *Cynanchum bungei* Decne. The structures of compounds including absolute configurations were elucidated by spectroscopic data and electronic circular dichroism (ECD) analysis. Anti-inflammatory activity evaluation revealed that compounds **1–7** inhibit the production of nitric oxide with IC_50_ values of 79.4, 49.7, 81.3, 40.2, 86.7, 90.1, and 54.4 μM, respectively, and decrease the levels of tumor necrosis factor-α, interleukin-6 contents in lipopolysaccharide-induced RAW264.7 macrophages.

## Introduction

The paxilline-type indole-terpenoids are one of the largest classes of fungal indole-terpenoids with diverse structures. Typical representatives of such compounds include paxilline (Springer et al., 1975), thiersinines (Li et al., 2002), lolicines (Munday-Finch et al., 1998), shearinines (Belofsky and Gloer, 1995), and penerpenes (Kong et al., 2019). Many of these compounds have significant bioactivities, such as antibacterial, and anti-inflammatory activities. Inflammation, as a protective response of living tissues to injury and infection and stress, involves a wide variety of physiological and pathological processes (Medzhitov, 2008). During the process, if acute inflammatory response fails to eliminate stimuli, it will devolve a chronic inflammation response, which is associated with many diseases, including asthma, cancer, stroke, and obesity (Zhong and Shi, 2019). The chronic inflammation response is characterized by secretion of nitric oxide (NO) and proinflammatory cytokines such as tumor necrosis factor-α (TNF-α) and interleukin-6 (IL-6) (Medzhitov, 2008). Therefore, finding novel and effective anti-inflammatory compounds is urgently required.

**FIGURE 1:**
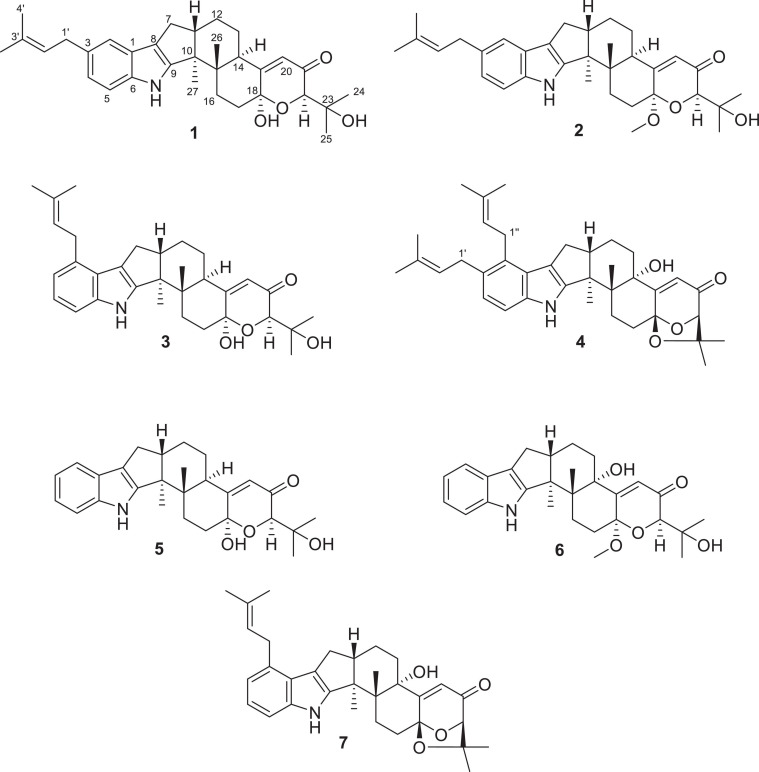
The chemical structures of compounds **1–7**.

In search of new compounds with anti-inflammatory activity, the secondary metabolites produced by *Penicillium* sp. HFF16 isolated from the rhizosphere soil of *Cynanchum bungei* Decne. in Mount Tai, East China, were investigated, which resulted in the isolation and identification of four new indole-terpenoids (**1–4**) named encindolene A, 18-*O*-methyl-encindolene A, encindolene B, and encindolene C, along with three known analogs including 7α-hydroxy-13-desoxy paxilline (**5**) (Peter and Christopher, 1994), 7-methoxypaxilline (**6**) (Ariantari et al., 2019), and paspalitrem C (**7**) (Dorner et al., 1984; Figure 1), which were isolated and identified. All of the compounds exhibited moderate inhibitory effects on the production of NO and proinflammatory cytokines (TNF-α and IL-6) in RAW264.7 macrophages stimulated by lipopolysaccharide (LPS). Herein, the isolation, structural elucidation, and bioactivities of these compounds were described.

## Materials and Methods

### General Experimental Procedures

Optical rotations were measured on a JASCO P-1020 digital polarimeter, and UV spectra were measured on a Beckman DU 640 spectrophotometer. Electronic circular dichroism (ECD) data were collected using a JASCO J-715 spectropolarimeter. NMR spectra were recorded on a Bruckmercury Plus-400 or a JNM-ECZR-500 spectrometers with TMS as an internal standard. High-resolution electrospray ionization mass spectrometry (HRESIMS) spectra were recorded with a Micromass Autospec-Uitima-TOF. Semipreparative high-performance liquid chromatography (HPLC) was carried out using an ODS column (YMC-pack ODS-A, 10 × 250 mm, 5 μm, 4 ml/min). Thin layer chromatography (TLC) and column chromatography (CC) were performed on plates precoated with silica gel GF_254_ (10–40 μm, Yantai Jiangyou Silicone Development Co., Ltd.).

### Fungal Material and Fermentation

The fungus *Penicillium* sp. HFF16 was isolated from the rhizosphere soil of *Cynanchum bungei* Decne. in Mount Tai, China, in May 2020. After grinding, the sample (1.0 g) was diluted to 10^–2^ g/ml with sterile H_2_O, 100 μl of which was deposited on Bengal red medium (maltose 20 g, monosodium glutamate 10 g, glucose 10 g, yeast extract 3 g, corn pulp 1 g, mannitol 20 g, sodium chloride 0.3 g, potassium dihydrogen phosphate 0.5 g, agar 20 g per liter of tap water) plate containing chloramphenicol (200 μg/ml) as a bacterial inhibitor. A single colony was transferred onto another PDA plate and was identified according to its morphological characteristics and ITS gene sequences (Supplementary Material). The data presented in the study are deposited in the GenBank, accessionnumber (MZ165618). A reference culture of *Penicillium* sp. HFF16 maintained at –80°C was deposited in our laboratory. The isolate was cultured on plates of PDA medium at 28°C for 4 days. Plugs of agar supporting mycelium growth were cut and transferred aseptically to 7 × 250-ml Erlenmeyer flasks each containing 100 ml of liquid medium (potato 200 g, glucose 20 g per liter of tap water) and cultured at 28°C at 150 RPM for 3 days. The seed liquid was inoculated aseptically into 140 × 1,000-ml Erlenmeyer flasks each containing rice medium (80 g rice, 100 ml tap water) at 0.5% inoculation amount and incubated at room temperature under static conditions for 35 days.

### Extraction and Isolation

The cultures (11.2 kg) were then extracted into EtOAc (40 L) by soaking overnight. The extraction was repeated three times. The combined EtOAc extracts were dried under vacuum to produce 38.2 g of extract. The EtOAc extract was subjected to a silica gel VLC column, eluting with a stepwise gradient of 0, 9, 11, 15, 20, 30, 50, and 100% EtOAc in petroleum ether (v/v) to give seven fractions (Fr. 1-7). Fraction 2 (2.3 g) was applied to ODS silica gel with gradient elution of MeOH-H_2_O (1:5, 2:3, 3:2, 4:1, and 1:0) to yield five subfractions (Fr. 2–1–Fr. 2–4). Fr. 2–4 (66 mg) was purified using semiprep HPLC (isocratic system 90% MeOH/H_2_O, v/v) to give compounds **7** (*t*_R_ 9.86 min; 14 mg) and **4** (*t*_R_ 16.41 min; 5.4 mg). Fraction 5 (7.3 g) was applied to ODS silica gel with gradient elution of MeOH-H_2_O (1:5, 2:3, 3:2, 4:1, and 1:0) to yield four subfractions (Fr. 5–1–Fr. 5–6). Fr. 5–1 (256 mg) was further purified using semiprep HPLC (isocratic system 90% MeOH/H_2_O, v/v) to give compound **5** (*t*_R_ 6.0 min; 4.7 mg). Fr. 5–2 (306 mg) was further purified using semiprep HPLC (isocratic system 90% MeOH/H_2_O, v/v) to give compounds **6** (*t*_R_ 8.4 min; 9.2 mg), **3** (*t*_R_ 10.5 min; 6.2 mg), and **1** (*t*_R_ 11.5 min; 5.3 mg). Fr. 5–3 (56 mg) was further purified using semiprep HPLC (isocratic system 90% MeOH/H_2_O, v/v) to give compound **2** (*t*_R_ 16.0 min; 3.7 mg).

*Encindolene A (****1***): white powder; [α]25 D–31 (*c* 0.1, MeOH); UV (MeOH) λ_max_ (log ε): 289 (2.85) and 237 (3.46) nm; ECD (0.25 mM, MeOH) λ_max_ 218 (–14.85), 242 (–8.04), 258 (+ 4.52), and 307 (+ 1.16) nm. ^1^H and ^13^C NMR data (Table 1); HRESIMS *m/z* 526.2902 [M + Na]^+^ (calcd for C_3__2_H_4__1_NO_4_Na, 526.2928).

**TABLE 1:** The ^1^H (400 MHz) and ^13^C NMR (100 MHz) data of compounds **1–3** in CD_3_OD.

**Position**	**1**		**2**		**3**	
	**δ_C_**	**δ_H_ (*J* in Hz)**	**δ_C_**	**δ_H_ (*J* in Hz)**	**δ_C_**	**δ_H_ (*J* in Hz)**
1	126.3, C		126.3, C		125.4, C	
2	118.0,CH	7.08, s	118.0, CH	7.07, s	133.5, C	
3	133.2, C		133.2, C		119.2, CH	6.70, d (8.0)
4	121.8, CH	6.81, d (7.9)	121.8, CH	6.80, d (7.9)	121.3, CH	6.88, t (8.0)
5	112.5, CH	7.18, d (7.9)	112.4, CH	7.18, d (7.9)	110.6, CH	7.12, d (8.0)
6	140.6, C		140.6, C		142.0, C	
7	28.1, CH_2_	2.34, dd (10.7, 12.9)	28.1, CH_2_	2.36, dd (10.7, 13.3)	30.2, CH_2_	2.51, dd (12.8, 14.9)
		2.65, dd (7.0, 12.9)		2.66, dd (7.0, 13.3)		2.82, overlap
8	117.8, C		117.8, C		117.5, C	
9	151.1, C		151.0, C		150.3, C	
10	51.6, C		51.6, C		51.4, C	
11	50.3, CH	2.74, m	50.4, CH	2.81, m	50.5, CH	2.85, overlap
12	25.3, CH_2_	1.75, overlap	25.4, CH_2_	1.80, overlap	25.3, CH_2_	1.81, m
		1.73, overlap		1.78, overlap		1.68, m
13	26.7, CH_2_	1.43, m	26.8, CH_2_	1.31, m	26.8, CH_2_	1.42, m
		1.61, m		1.64, m		1.71, m
14	43.3, CH	2.80, m	43.4, CH	2.80, m	43.3, C	2.84, m
15	43.8, C		43.5, C		43.9, C	
16	31.9, CH_2_	1.88, m	31.4, CH_2_	1.88, m	31.8, CH_2_	1.952, m
		2.23, m		2.04, m		2.25, m
17	37.1, CH_2_	2.13, m	29.9, CH_2_	2.49, m	37.1, CH_2_	2.15, m
		2.13, m		1.90, m		2.15, m
18	95.0, C		98.1, C		95.0, C	
19	168.1, C		167.0, C		168.1, C	
20	122.7, CH	5.71, s	122.8, CH	5.74, s	122.7, CH	5.74, s
21	199.8, C		198.6, C		199.8, C	
22	78.2, CH	4.20, s	78.5, CH	4.02, s	78.2, CH	4.20, s
23	73.4, C		73.2, C		73.4, C	
24	25.2, CH_3_	1.27, s	25.4, CH_3_	1.30, s	25.2, CH_3_	1.27, s
25	26.6, CH_3_	1.28, s	26.3, CH_3_	1.30, s	26.6, CH_3_	1.28, s
26	15.7, CH_3_	0.91, s	15.9, CH_3_	0.99, s	15.7, CH_3_	0.98, s
27	14.8, CH_3_	1.07, s	14.8, CH_3_	1.07, s	14.8, CH_3_	1.09, s
27–OCH_3_			49.6, CH_3_	3.41, s		
1′	35.5, CH_2_	3.36, d (7.4)	35.5, CH_2_	3.36, d (7.0)	31.8, CH_2_	3.56, d (7.4)
2′	126.2, CH	5.36, t (7.4)	126.3, CH	5.35, t (7.4)	125.6, CH	5.35, t (7.4)
3′	131.8, C		131.8, C		132.1, C	
4′	17.9, CH_3_	1.74, overlap	17.9, CH_3_	1.74, overlap	18.1, CH_3_	1.76, s
5′	26.0, CH_3_	1.74, overlap	26.0, CH_3_	1.74, overlap	25.9, CH_3_	1.73, s

*18-O-methyl-encindolene A (****2***): white powder; [α]25 D–25 (*c* 0.1, MeOH); UV (MeOH) λ_max_ (log ε): 284 (2.89) and 237 (3.51) nm; ECD (0.24 mM, MeOH) λ_max_ 216 (–12.32), 243 (–9.41), 259 (+ 2.75), and 308 (+ 1.35) nm. ^1^H and ^13^C NMR d*18-O-methyl-encindolene A (****2)***: white powder; [α]25 D–25 (*c* 0.1, MeOH); UV (MeOH) λ_max_ (log ε): 284 (2.89) and 237 (3.51) nm; ECD (0.24 mM, MeOH) λ_max_ 216 (–12.32), 243 (–9.41), 259 (+ 2.75), and 308 (+ 1.35) nm. ^1^H and ^13^C NMR data (Table 1); ^1^H and ^13^C NMR data (Table 1); HRESIMS *m/z* 540.3078 [M + Na]^+^ (calcd for C_3__3_H_4__3_NO_4_Na, 540.3084).

*Encindolene B (****3***): white powder; [α]25 D–16 (*c* 0.1, MeOH); UV (MeOH) λ_max_ (log ε): 287 (2.73) and 235 (3.31) nm; ECD (1.2 mM, MeOH) λ_max_ 2*Encindolene B (****3)***: white powder; [α]25 D–16 (*c* 0.1, MeOH); UV (MeOH) λ_max_ (log ε): 287 (2.73) and 235 (3.31) nm; ECD (1.2 mM, MeOH) λ_max_ 216 (–9.10), 239 (–11.19), 258 (+ 2.27), and 286 (+ 0.94) nm. ^1^H and ^13^C NMR data (Table 1); HRESIMS *m/z* 526.2903 [M + Na]^+^ (calcd for C_3__2_H_4__1_NO_4_Na, 526.2928).

*Encindolene C (****4***): white powder; [α]25 D + 98 (*c* 0.1, MeOH); UV (MeOH) λ_max_ (log ε): 285*Encindolene C (****4)***: white powder; [α]25 D + 98 (*c* 0.1, MeOH); UV (MeOH) λ_max_ (log ε): 285 (3.02) and 237 (3.54) nm; ECD (0.22 mM, MeOH) λ_max_ 207 (+ 13.75), 245 (–31.6), 274 (+ 9.23), and 357 (+ 7.98) nm. ^1^H and ^13^C NMR data (Table 2); HRESIMS *m/z* 570.3581 [M + H]^+^ (calcd for C_3__7_H_4__8_NO_4_Na, 570.3578).

**TABLE 2:** The ^1^H (400 MHz) and ^13^C NMR (100 MHz) data of compound **4** in CD_3_OD.

**Position**	**4**	
	**δ_C_**	**δ_H_ (*J* in Hz)**
1	126.6, C	
2	130.7,C	
3	130.4, C	
4	123.0, CH	6.75, d (7.9)
5	110.6, CH	7.05, d (7.9)
6	140.6, C	
7	30.6, CH_2_	2.48, dd (11.3, 12.9)
		2.75, overlap
8	116.3, C	
9	153.4, C	
10	52.5, C	
11	50.1, CH	2.79, m
12	22.3, CH_2_	2.04, overlap
		1.94, overlap
13	26.7, CH_2_	2.62, m
		1.90, m
14	77.9, C	
15	40.8, C	
16	27.6, CH_2_	1.88, m
		2.23, m
17	29.4, CH_2_	2.82, m
		1.99, m
18	106.2, C	
19	172.4, C	
20	118.2, CH	5.80, s
21	199.4, C	
22	89.1, CH	4.29, s
23	79.4, C	
24	23.4, CH_3_	1.13, s
25	29.2, CH_3_	1.41, s
26	23.8, CH_3_	1.22, s
27	16.6, CH_3_	1.36, s
1′	32.3, CH_2_	3.31, overlap
2′	126.5, CH	5.22, t (7.2)
3′	130.7, C	
4′	18.0, CH_3_	1.72, s
5′	26.0, CH_3_	1.70, s
1″	30.1, CH_2_	3.55, m
2″	126.4, CH	5.11, t (7.2)
3″	131.1, C	
4″	18.3, CH_3_	1.78, s
5″	25.9, CH_3_	1.68, s

1.68,make sure all Supplementary Files are cited. Please also provide captions for these files, if relevant. Note that ALL Supplementary Files will be deposited to FigShare and receive a DOI. Notify us of any previously deposited material. Please provide the meaning of “*,#” specified in Figure 5.

Cell viability of the test compounds were detected using MTT assay (Pan et al., 2021). RAW264.7 cells (Type Culture Collection of the Chinese Academy of Sciences, Shanghai, China) were cultured in DMEM supplemented with 10% fetal bovine serum (Gibco, United States) at 37°C in a 5% CO_2_ incubator. Cells were seeded in a 96-well plate at a concentration of 8 × 10^5^ cells/well and treated with LPS (5 μg/ml) and various concentrations of test compounds (1–200 μM) for 24 h. After that, MTT solution (10 μl) was added and incubated at 37°C for 4 h. The purple crystals dissolved with dimethylsulfoxide (150 μl) were added, and the absorbance value was measured by a microplate reader at 570 nm.

### Measurement of NO, TNF-α, and IL-6 Production

RAW264.7 cells were seeded in a 96-well plate at a concentration of 8 × 10^5^ cells/well. After incubation, cells were pretreated with the test compounds with different dose (20–160 μM) and then stimulated with LPS (5 μg/ml) for 24 h. The NO concentration in culture medium was calculated by the commercial kit (Jiancheng, Nanjing, China) according to the manufacturer’s instruction. The TNF-α and IL-6 levels were determined using ELISA (Xiao et al., 2017). In brief, the cells were incubated with the test compounds (50 μM) in the presence or absence of LPS (5 μg/ml). After incubation for 24 h, the supernatant was detected for TNF-α and IL–6 at 450 nm. All data were expressed as the mean ± SD from at least three independent experiments.

## Results and Discussion

### Structure Elucidation of Compounds

**FIGURE 2:**
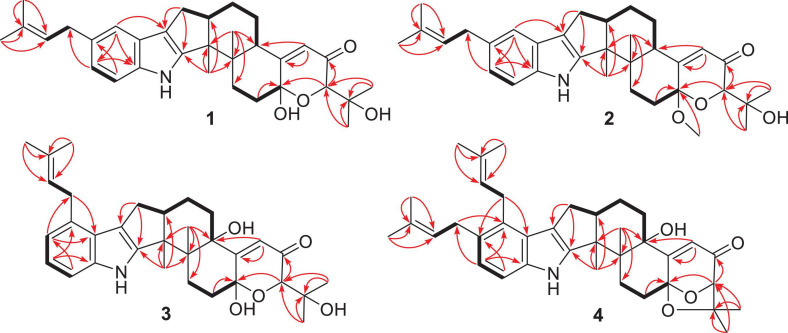
Selected HMBC and COZY correlations of **1–4**.

**FIGURE 3:**
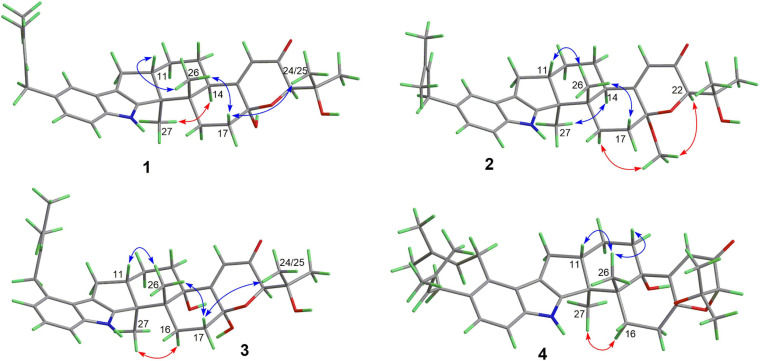
Selected NOESY correlations of **1–4**.

**FIGURE 4:**
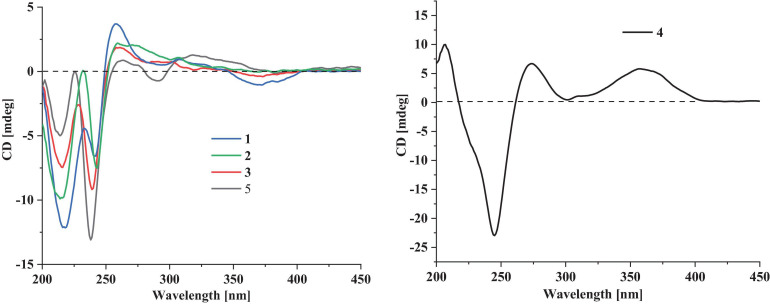
The experimental ECD curves of **1–5**.

Compound **1** has a molecular formula C_3__2_H_4__1_NO_4_ as established from its HRESIMS and ^13^C NMR data (Supplementary Table 1). The ^1^H and ^13^C NMR data of **1** (Table 1), with the aid of a heteronuclear single quantum coherence (HSQC) spectrum, showed a total of 32 carbon signals comprising 1 ketone carbonyl, 12 olefinic or aromatic carbons with 5 protonated, 6 sp^3^ methylenes, 3 sp^3^ methines with 1 oxygenated, 4 sp^3^ non-protonated carbons with 2 oxygenated, and 6 methyls. These data are quite similar to those of 7α-hydroxy-13-desoxy paxilline (**5**) with the main differences being the presence of additional signals (δ_C/H_ 35.3/3.36, 126.2/5.36, 131.8, 17.9/1.74, and 26.0/1.74) corresponding to an isopentene group in the NMR data of **1**. Besides, unlike that of **5**, only three aromatic protons resonating into an ABX system were observed in the ^1^H NMR data of **1**, indicating that the C–3 or C–4 of **1** was substituted. The above data suggested that **1** was a prenylated derivative of **5**. COZY correlations (Figure 2) of H_2_-1′/H-2′ as well as HMBC correlations (Figure 2) from H_3_-4′ and H_3_-5′ to C-3′ and C-2′ and from H_2_–1′ to C–2, C–3, and C–4 confirmed the presence of an isopentene group at C–3. The remaining substructure was deduced to be the same as that of **5** by their similar NMR chemical shifts, which was further corroborated by detailed analysis of the two-dimensional NMR data (Figure 2) of **1**. NOESY correlations (Figure 3) of H_3_–24(25)/H–17/H_3_–26/H–11 suggested the same face of these protons, while NOESY correlation of H_3_–27/H–14 indicated that they were on the face opposite to H_3_–26. The absolute configuration of **1** was determined to be the same as that of **5** by their similar ECD curves (Figure 4). Accordingly, compound **1** was assigned as a new indole-terpenoid and named as encindolene A.

**FIGURE 5:**
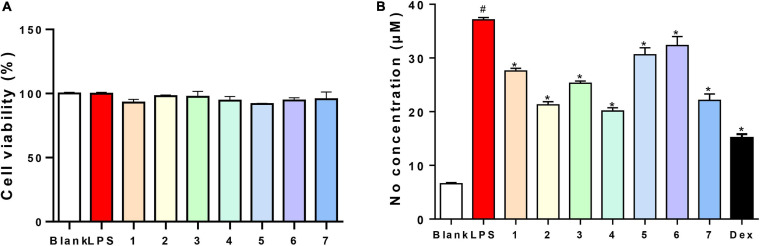
Viability effects and NO inhibitory activities of compounds 1–7 **(A)**: 200 μM; **(B)**: 40 μM) on RAW264.7 cells.

Compound **2** was determined to have the molecular formula C_3__3_H_4__3_NO_4_ based on the positive HRESIMS data, containing an additional methyl substituent in comparison with **1**. The NMR spectra of **2** were closely related to those of **1** except for the appearance of an additional methoxy group at δ_C/H_ 49.6/3.41. The location of this methoxy group at C-18 in **2** was confirmed by the HMBC correlation (Figure 2) from its protons to C-18 (δ_C_ 98.1). Thus, compound **2** was elucidated as 18-*O*-methyl-sperindolene A according to compound **1**. The relative and absolute configurations of **2** were determined to be the same as **1** by NOESY correlations (Figure 3) of H-22/MeO-18/H-16, H_3_–27/H–14, and H–11/H_3_–26/H–17, as well as the comparison of the ECD curve of **1** with that of **2** (Figure 4).

Compound **3** possessed the molecular formula C_3__2_H_4__1_NO_5_ as determined by HRESIMS data. The ^1^H-NMR, ^13^C-NMR, and HSQC data of **3** were quite similar to those of **1**. However, three continuous proton signals at δ_H_ 6.70, 6.88, and 7.12 instead of an ABX coupling system as in **1** were shown in the aromatic region of the ^1^H-NMR of **3**, implying the position of the isopentene group at C-2 or C-5. HMBC correlations (Figure 2) from H-1′ to C-1, C-2, and C-3 demonstrated the position of the isopentene group at C-2. The relative configuration of 3 was assigned to be the same as that of **1** by ROESY correlations (Figure 3) of H–24(25)/H–17/H_3_–26/H–11 and H_3_–27/H–14. The ECD curve (Figure 4) of **3** is very similar to those of **1** and **2**, leading to the assignment of the absolute configuration of **3** as shown in Figure 1.

The HRESIMS data for **4** showed an ion peak at *m*/*z* 570.3581 [M + H]^+^, indicating the molecular formula C_3__7_H_4__7_NO_4_. The UV absorption at 237 and 285 nm suggested that **4** was also an indole-terpenoid. The ^13^C NMR data of **4** are very similar to that of paspalitrem C (**7**) (Dorner et al., 1984) except for the presence of five additional signals at δ_C/H_ 30.1/3.55, 126.4/5.11, 131.1, 18.3/1.78, and 25.9/1.68 corresponding to an isopentene group, as deduced from HMBC correlations from H_3_–4″ and H_3_–5″ to C-3″ and C-2″ and COZY correlations of H-2″ and H_2_–1″. Besides, unlike that of compound 7, the ^1^H NMR spectrum of **4** showed the presence of only two proton signals (δ_H_ 6.75 and 7.05) in the aromatic region, which are coupled to each other. This suggested the location of the above isopentene group at C-2. HMBC correlations (Figure 2) from H_2_–1″ to C-1, C-2, and C-3 further corroborated this deduction. The remaining substructure was determined to be the same as that of **7** by detailed analysis of the HMBC and COSY data (Figure 2). The relative configuration of compound **4** was also deduced to be the same as that of **7** by their nearly identical 1D NMR chemical shifts of C-7 to C-27 and was further confirmed by NOE correlations (Figure 3) of H–11/H_3_–26/H–13 and H_3_–27/H–16. The ECD curve of **4** showed strong positive Cotton effects (CEs) around 210, 275, and 325 nm (Figure 4). These data were very similar to those of shearilicine (Ariantari et al., 2019), a previously described analog bearing similar carbon skeleton as that of **4**, thus leading to the assignment of the absolute configuration of **4**.

### Anti-inflammatory and Antibacterial Activities Assay

**FIGURE 6:**
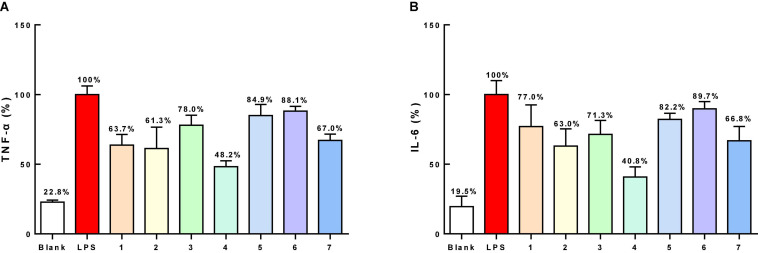
The inhibitory effects on TNF-α **(A)** and IL-6 **(B)** production of compounds 1–7 (50 μM) on RAW264.7 cells.

The antibacterial activities of all of the isolated compounds against *Staphylococcus aureus* and *Escherichia coli* were evaluated using the twofold dilution assay (Song et al., 2021). The results showed that all of the compounds were inactive. Compounds **1–7** were also non-cytotoxic to RAW264.7 cells at the concentration of 200 μM by MTT assay (Figure 5A). NO production was used as an indicator to evaluate the anti-inflammatory activity of **1–7**. All of the compounds showed varying degrees of inhibitory activities on the production of NO in LPS-stimulated RAW264.7 cells, with IC_50_ values of 79.4, 49.7, 81.3, 40.2, 86.7, 90.1, and 54.4 μM, respectively, while 13.30 μM for dexamethasone, a positive control. Based on this, compounds **1–7** (40 μM) could suppress NO overproduction in cells (Figure 5B). To further confirm the anti-inflammatory activities, the effects of compounds **1–7** on the production of proinflammatory cytokines (TNF-α and IL-6) in LPS-induced RAW264.7 cells were evaluated. All the compounds showed moderate inhibitory effects on the production of TNF-α and IL-6 production at the concentration of 50 μM, with compound **4** showing the strongest effect (Figure 6).

## Conclusion

In summary, from the fungus *Penicillium* sp. HFF16, seven indole-terpenoids including four new were isolated and identified. These compounds could inhibit NO, TNF-α, and IL-6 production without affecting the cell viability in LPS-stimulated RAW264.7 macrophages. These results further demonstrated that fungi from medicinal plants are an abundant source of new bioactive products with medicinal use.

## Data Availability Statement

The datasets presented in this study can be found in online repositories. The names of the repository/repositories and accession number(s) can be found below: GenBank, MZ165618.

## Author Contributions

GP conceived and designed the experiments and was involved in isolation of compounds. YZ, SR, FL, QX, and XZ contributed to isolation of compounds. WP contributed to the collection of physical data of compounds. MY, CP, and GH performed genetic manipulation, strain fermentation, and extraction. TY contributed to the collection of the NMR data of compounds. FK revised the manuscript. LZ supervised the work and prepared the manuscript. NX contributed to bioactivity assay and revised the manuscript. All authors contributed to the article and approved the submitted version.

## Conflict of Interest

The authors declare that the research was conducted in the absence of any commercial or financial relationships that could be construed as a potential conflict of interest.
