# Supplementary material for: Indole-Terpenoids With Anti-inflammatory Activities From Penicillium sp. HFF16 Associated With the Rhizosphere Soil of Cynanchum bungei Decne
Source: Front Microbiol. 2021 Jul 9;12:710364. doi: 10.3389/fmicb.2021.710364 (PMC8302409; doi:10.3389/fmicb.2021.710364)

Supplementary Material

Indole-terpenoids with anti-inflammatory activities from *Penicillium* sp. HFF16 associated with the rhizosphere soil of *Cynanchum bungei* Decne.

Guojun Pan,[1] Yanfen Zhao,[1] Shuang Ren,[1] Fengyang Liu,[1] Qicai Xu,[1] Weibin Pan,[2] Tongtao Yang, [3] Mingtian Yang,[1] Xinru Zhang,[1] Chuanyue Peng,[1] Gangping Hao,[1] Fandong Kong, [2] Liman Zhou,[2*] Na Xiao [3, 4 *]

[1] *College of Life Sciences, Shandong First Medical University & Shandong Academy of Medical Sciences, Tai'an, Shandong 271000, China.*
[2] *Key Laboratory of Chemistry and Engineering of Forest Products, State Ethnic Affairs Commission, Guangxi Key Laboratory of Chemistry and Engineering of Forest Products, Guangxi Collaborative Innovation Center for Chemistry and Engineering of Forest Products, School of Chemistry and Chemical Engineering, Guangxi University for Nationalities, Nanning 530006, China.*
[3] *State Key Laboratory of Crop Biology, college of Agronomy, Shandong Agriculture University, Tai'an, Shandong 271018, China.*
[4] *State Key Laboratory of Natural Medicines, China Pharmaceutical University, Nanjing 210009, China.*

List of Supporting Information

The ITS gene sequences of *Aspergillus* sp. CYH26……………………………………….S3
Figure S1 The picture of *Penicillium* sp. HFF16...S3.
Figure S2 ^{1}H-NMR spectrum of **1** in CD_3OD...S4.
Figure S3. ^{13}C-NMR spectrum of **1** in CD_3OD...S4.
Figure S4. DEPT spectrum of **1** in CD_3OD...S5.
Figure S5. HSQC spectrum of **1** in CD_3OD..S5.
Figure S6. COSY spectrum of **1** in CD_3OD..S6.
Figure S7. HMBC spectrum of **1** in CD_3OD..S6.
Figure S8. NOESY spectrum of **1** in CD_3OD...S7.
Figure S9. HRESIMS spectrum of **1** in CD_3OD...S7.
Figure S10. ^{1}H-NMR spectrum of **2** in CD_3OD..S8.
Figure S11. ^{13}C-NMR spectrum of **2** in CD_3OD..S8.
Figure S12. DEPT spectrum of **1** in CD_3OD..S9.
Figure S13. HSQC spectrum of **2** in CD_3OD...S9.
Figure S14. COSY spectrum of **2** in CD_3OD...S10.
Figure S15. HMBC spectrum of **2** in CD_3OD..S10.

Figure S16. NOESY spectrum of **2** in CD_3OD........S11.
Figure S17. HRESIMS spectrum of **2** in CD_3OD........S11.
Figure S18. ^{1}H-NMR spectrum of **3** in CD_3OD........S12.
Figure S19. ^{13}C-NMR spectrum of **3** in CD_3OD........S12.
Figure S20. DEPT spectrum of **1** in CD_3OD........S13.
Figure S21. HSQC spectrum of **3** in CD_3OD........S13.
Figure S22. COSY spectrum of **3** in CD_3OD........S14.
Figure S23. HMBC spectrum of **3** in CD_3OD........S14.
Figure S24 NOESY spectrum of **3** in CD_3OD........S15.
Figure S25. HRESIMS spectrum of **3** in CD_3OD........S15.
Figure S26. ^{1}H-NMR spectrum of **4** in CD_3OD........S16.
Figure S27. ^{13}C-NMR spectrum of **4** in CD_3OD........S16.
Figure S28. DEPT spectrum of **1** in CD_3OD........S17.
Figure S29. HSQC spectrum of **4** in CD_3OD........S17.
Figure S30. COSY spectrum of **4** in CD_3OD........S18.
Figure S31 HMBC spectrum of **4** in CD_3OD........S18.
Figure S32. NOESY spectrum of **4** in CD_3OD........S19.
Figure S33. HRESIMS spectrum of **4** in CD_3OD........S19.

The ITS sequence of *Penicillium sp.* HFF16

CCTGATCCGAGGTCACCTGGAAAGATTGATTGGGGTCGCCGGCGGGC
GCCGGCCGGGCCTACAGAGCGGGTGACGAAGCCCCATACGCTCGAGG
ACCGGACGCGGTGCCGCCGCTGCCTTTCGGGCCCGCCCCCCCGGGG
GGAGGGGCGGGGGCCCAACACACAAGCCGTGCTTGAGGGCAGCAAT
GACGCTCGGACAGGCATGCCCCCCGGAATACCAGGGGGCGCAATGTG
CGTTCAAAGACTCGATGATTCACTGAATTCTGCAATTCACATTACTTAT
CGCATTTCGCTGCGTTCTTCATCGATGCCGGAACCAAGAGATCCGTTG
TTGAAAGTTTTAACTGATTTAGCTAATCGCTCAGACTGCAATCTTCAGA
CAGAGTTCAATGGTGTCTTCGGCGGGCGCGGGCCCGGGGGCGGGTG
CCCCCCGGCGGCCGTGAGGCGGGCCCGCCGAAGCAACAAGGTACGAT
AAACACGGGTGGGAGGTTGGACCCAGAGGGCCCTCACTCGGTAATGA
TCCTTCCGCAGGTCACCCTACGGAAGGCTAC

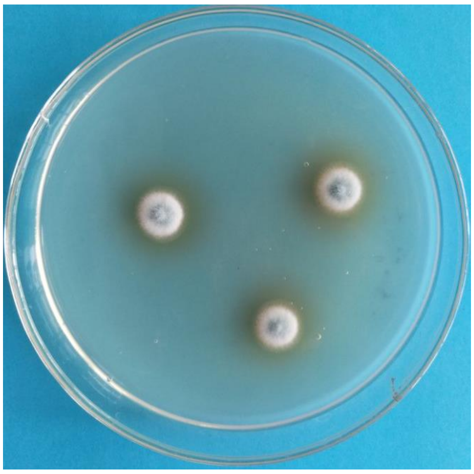

Figure S1. The picture of *Penicillium sp.* HFF16

Figure S2. ^{1}H-NMR spectrum of 1 in CD_3OD

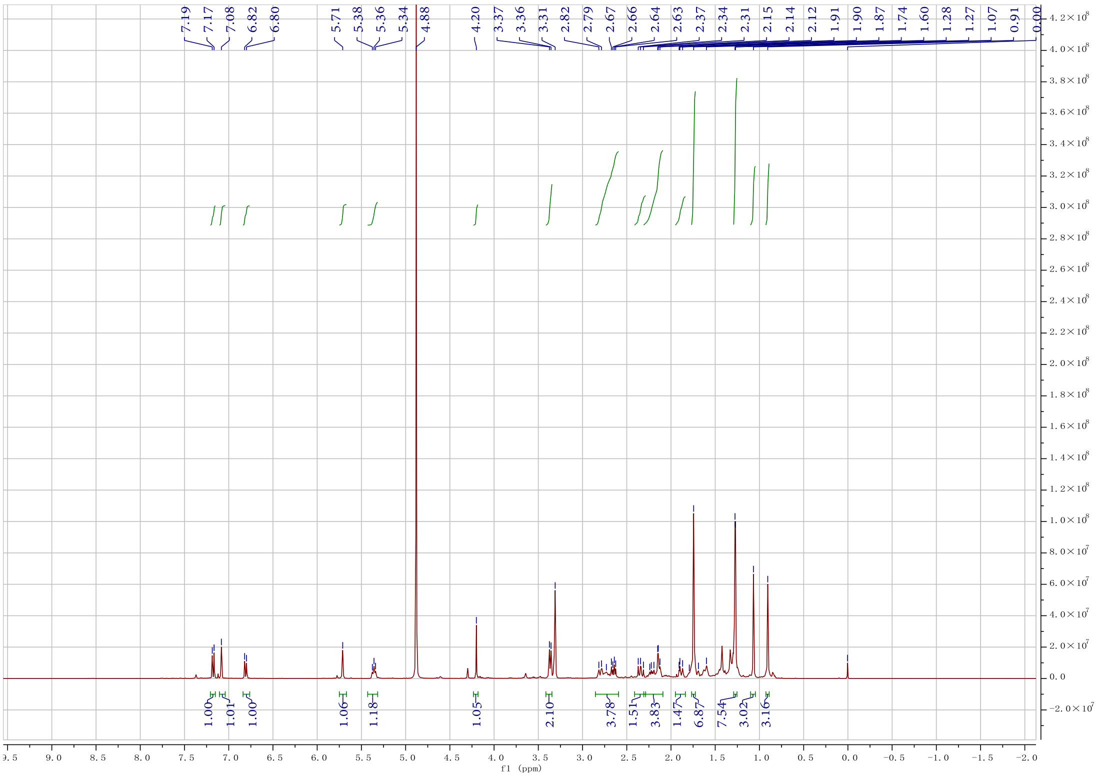

Figure S3. ^{13}C-NMR spectrum of 1 in CD_3OD

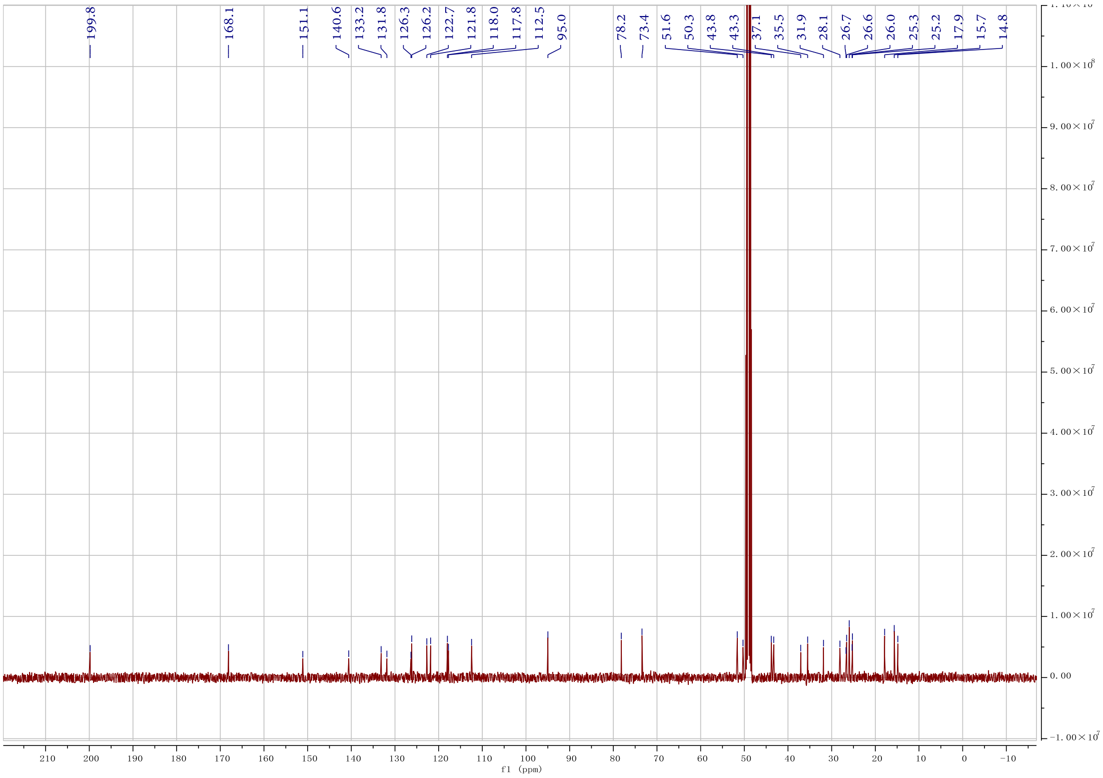

Figure S4. DEPT spectrum of 1 in CD_3OD

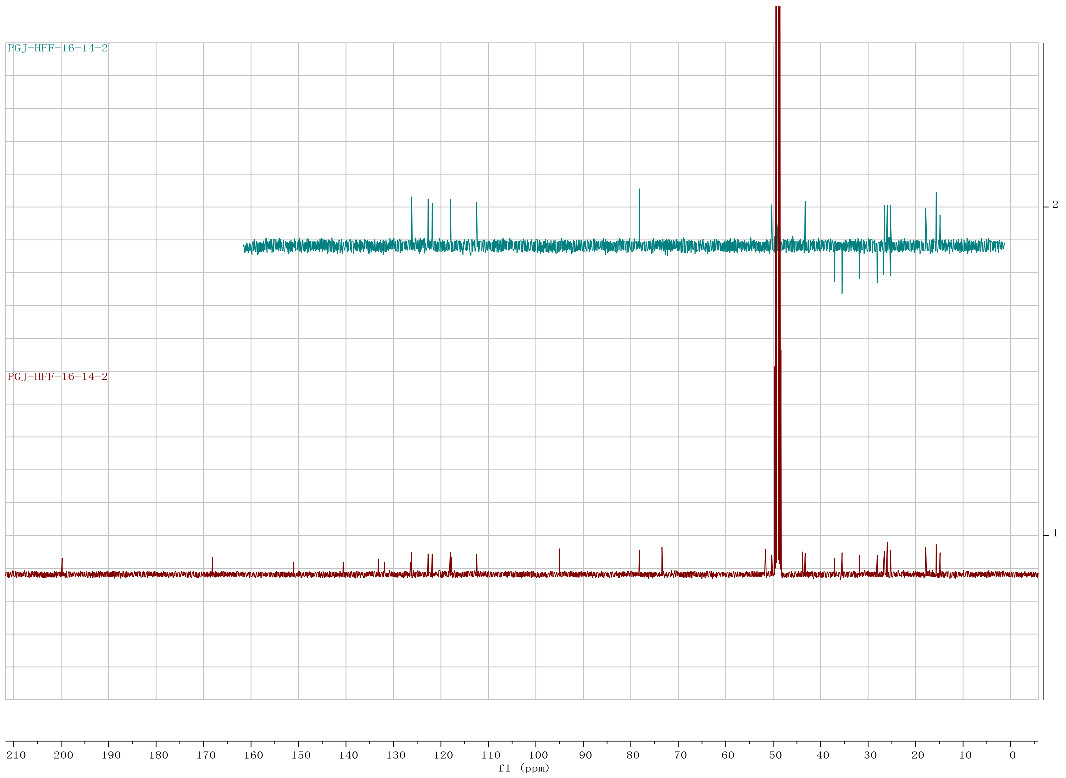

Figure S5. HMQC spectrum of 1 in CD_3OD

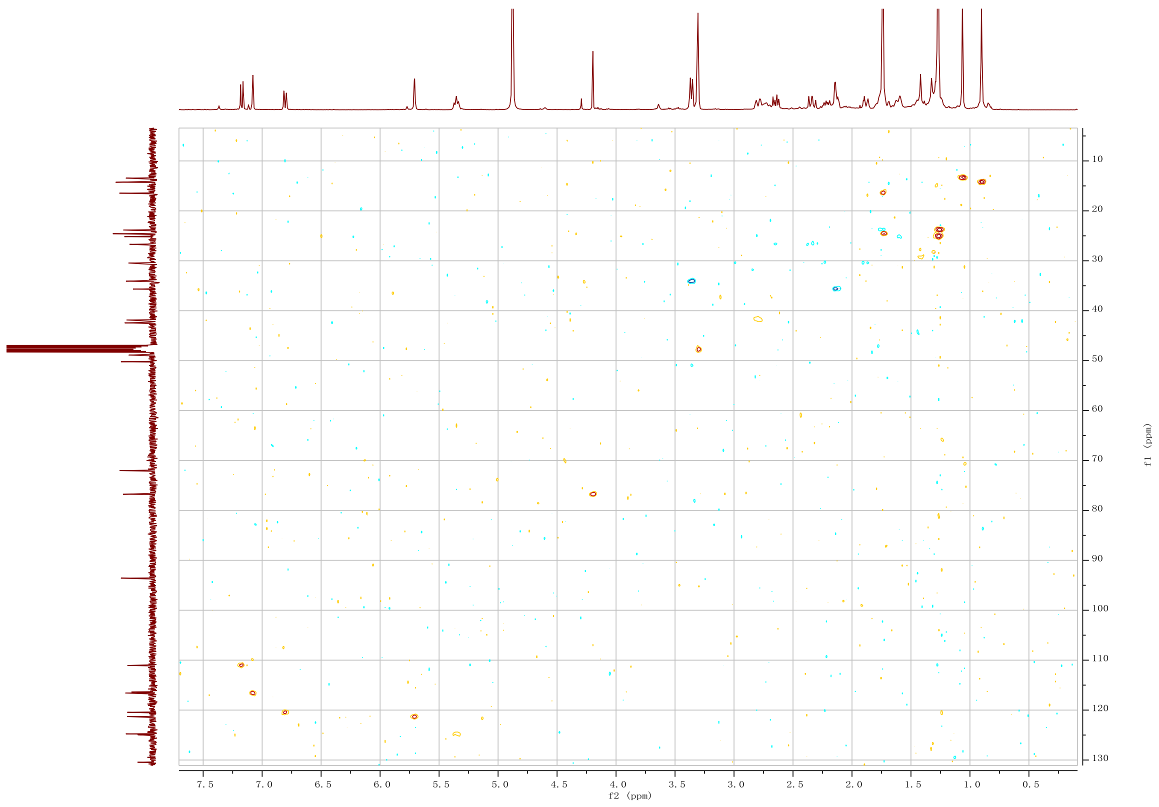

Figure S6. ^{1}H-^{1}H COSY spectrum of 1 in CD_3OD

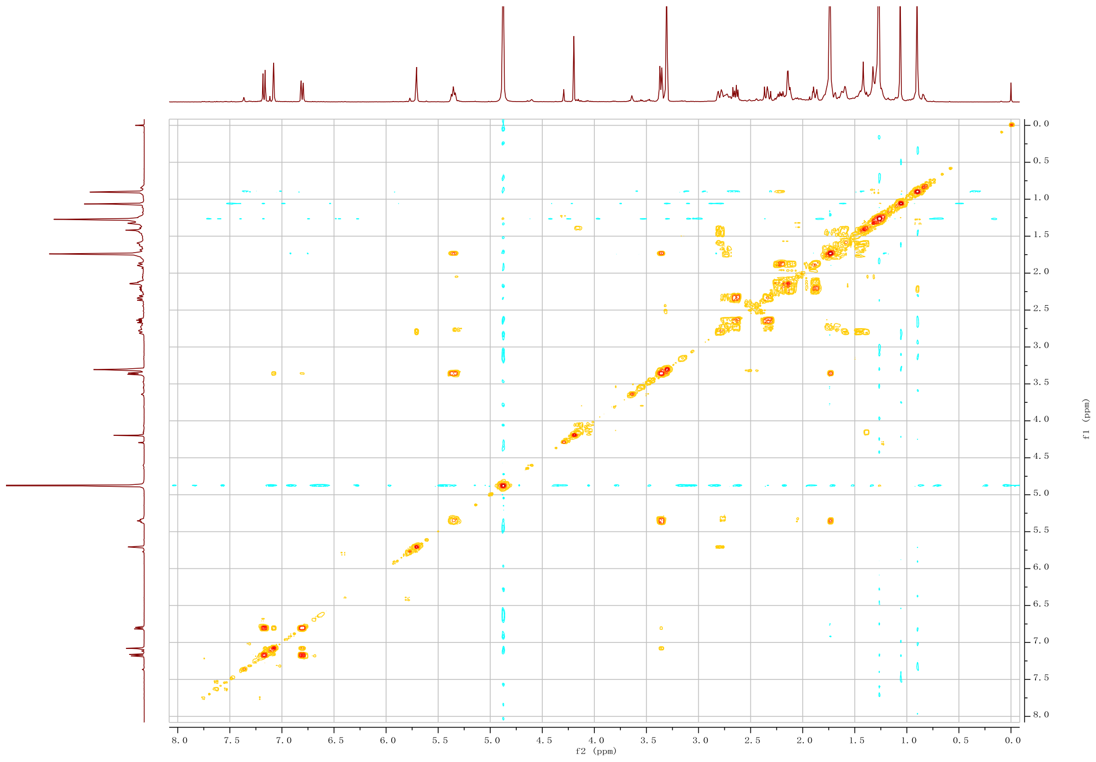

Figure S7. HMBC spectrum of 1 in CD_3OD

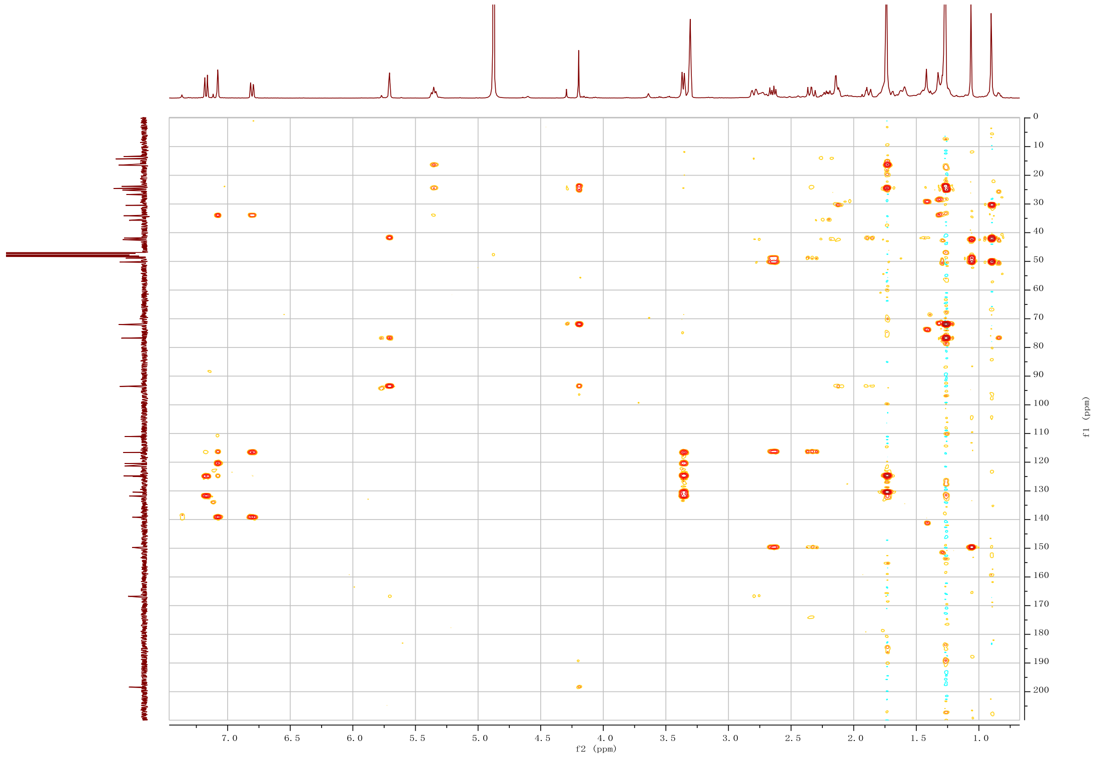

Figure S8. NOESY spectrum of 1 in CD_3OD

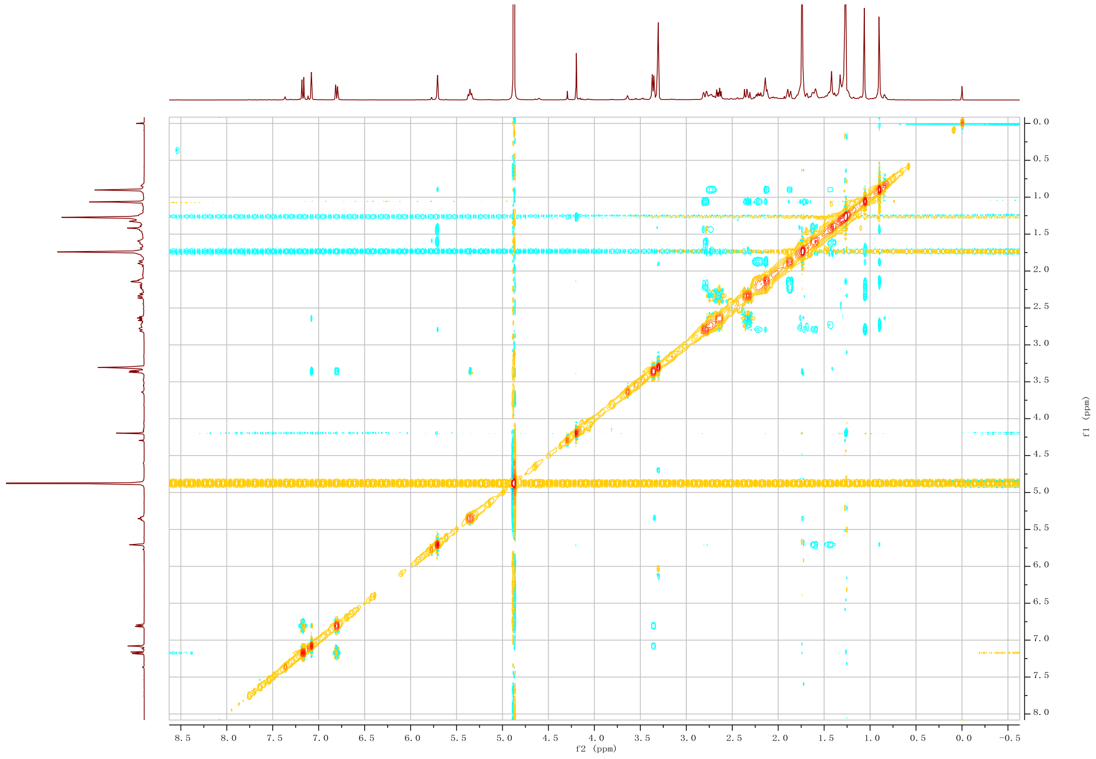

Figure S9. HRESIMS spectrum of 1

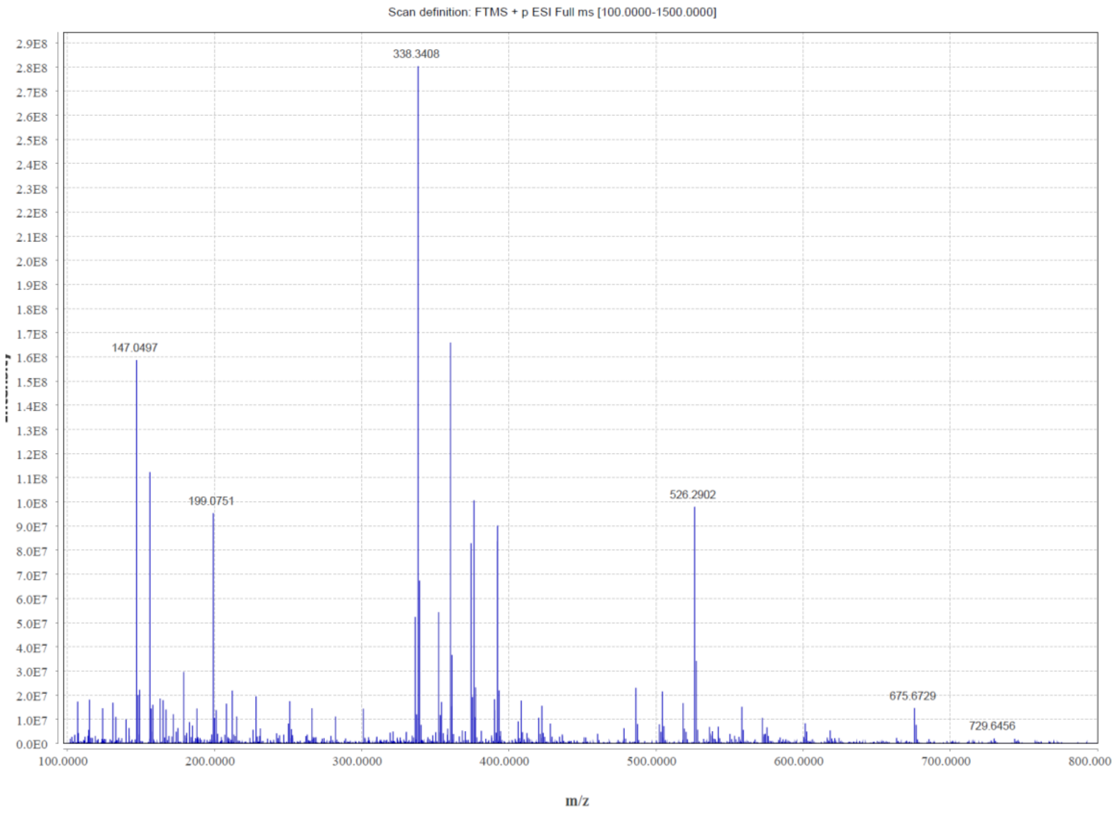

Figure S10. ^{1}H-NMR spectrum of 2 in CD_3OD

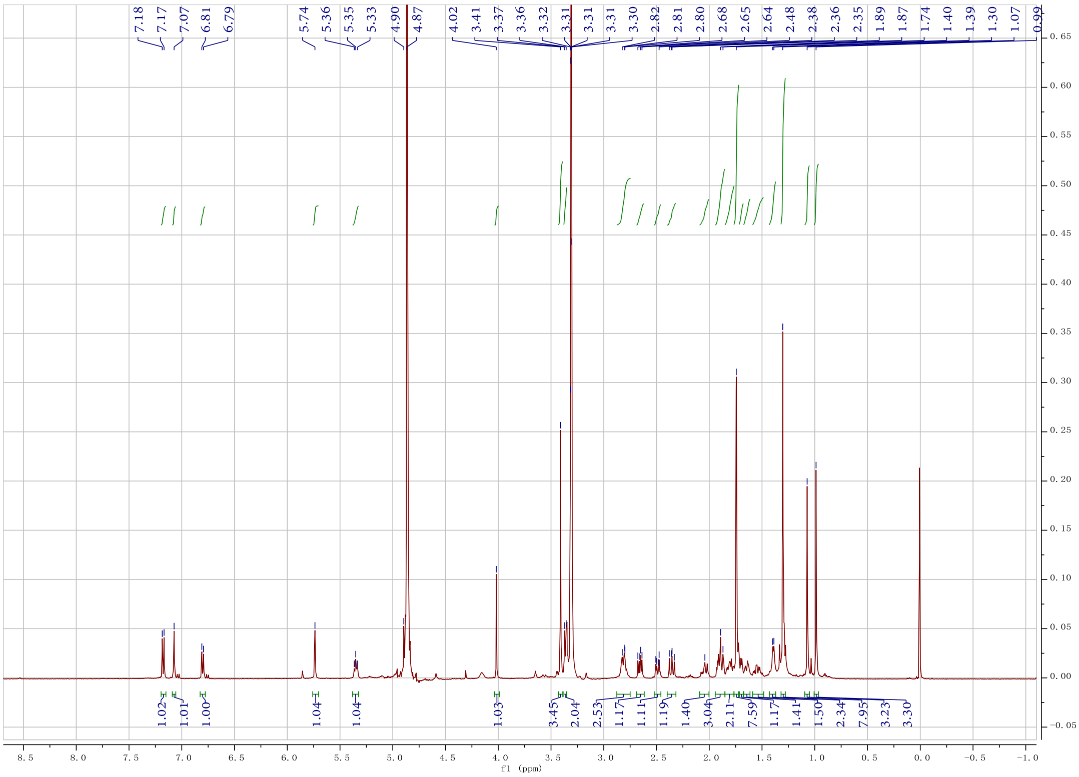

Figure S11. ^{13}C-NMR spectrum of 2 in CD_3OD

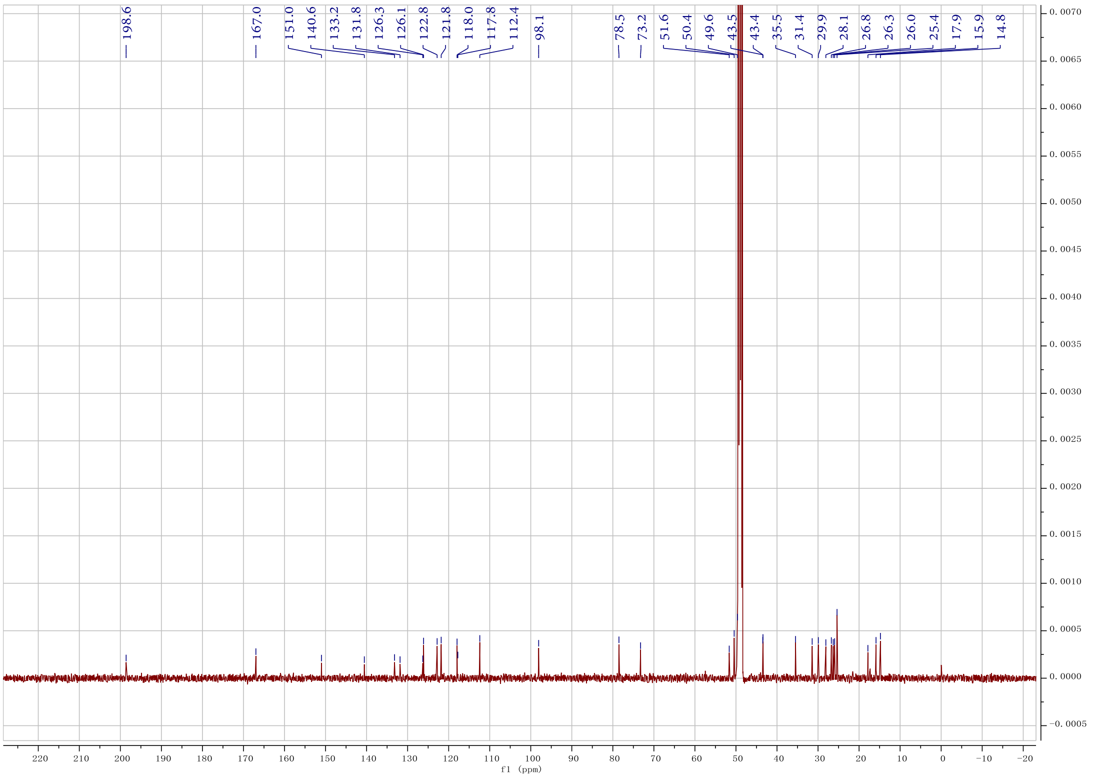

Figure S12. DEPT spectrum of 2 in CD_3OD

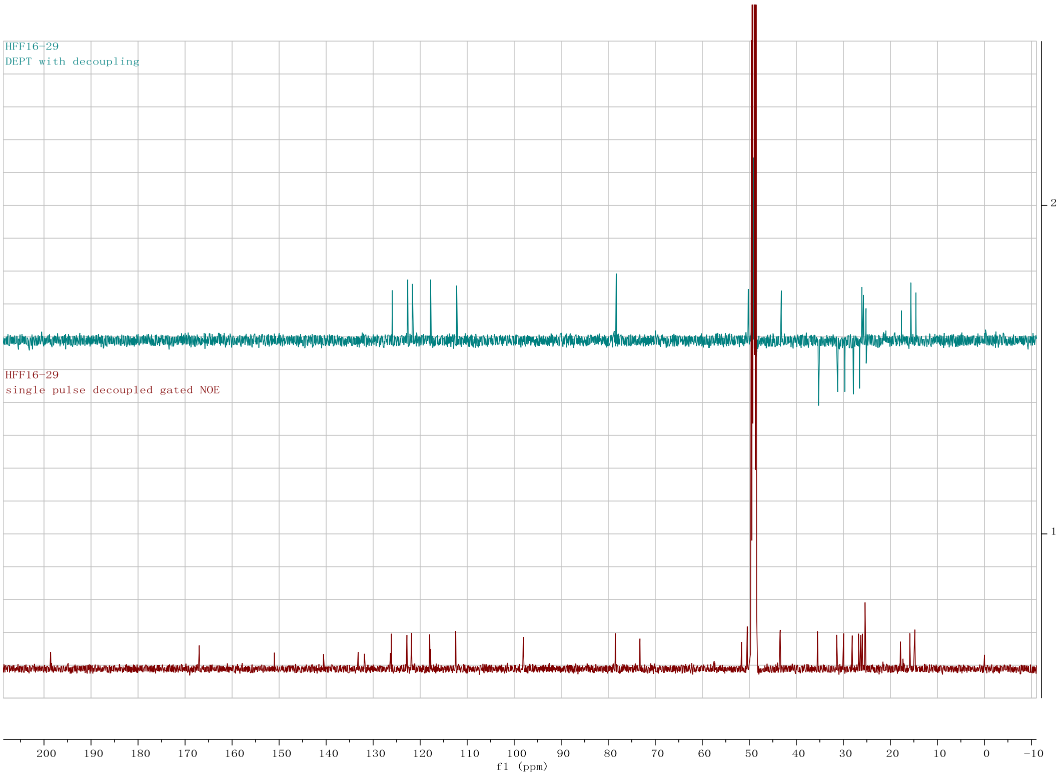

Figure S13. HSQC spectrum of 2 in CD_3OD

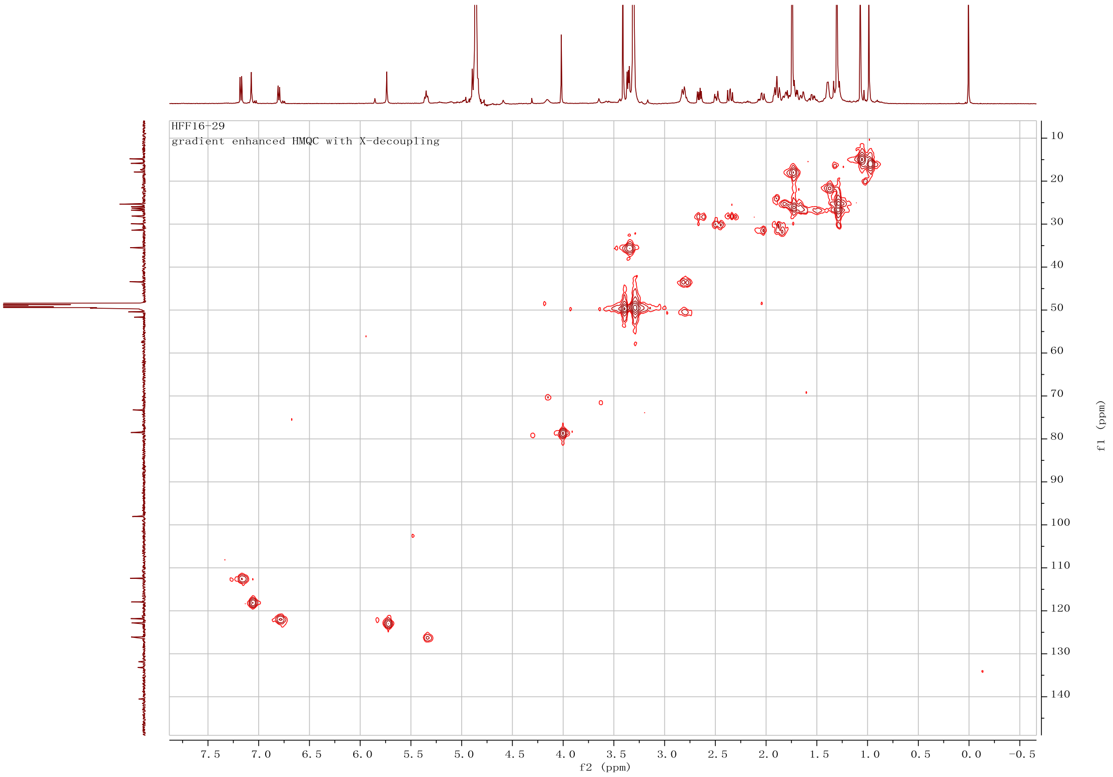

Figure S14. ^1H-^1H COSY spectrum of 2 in CD_3OD

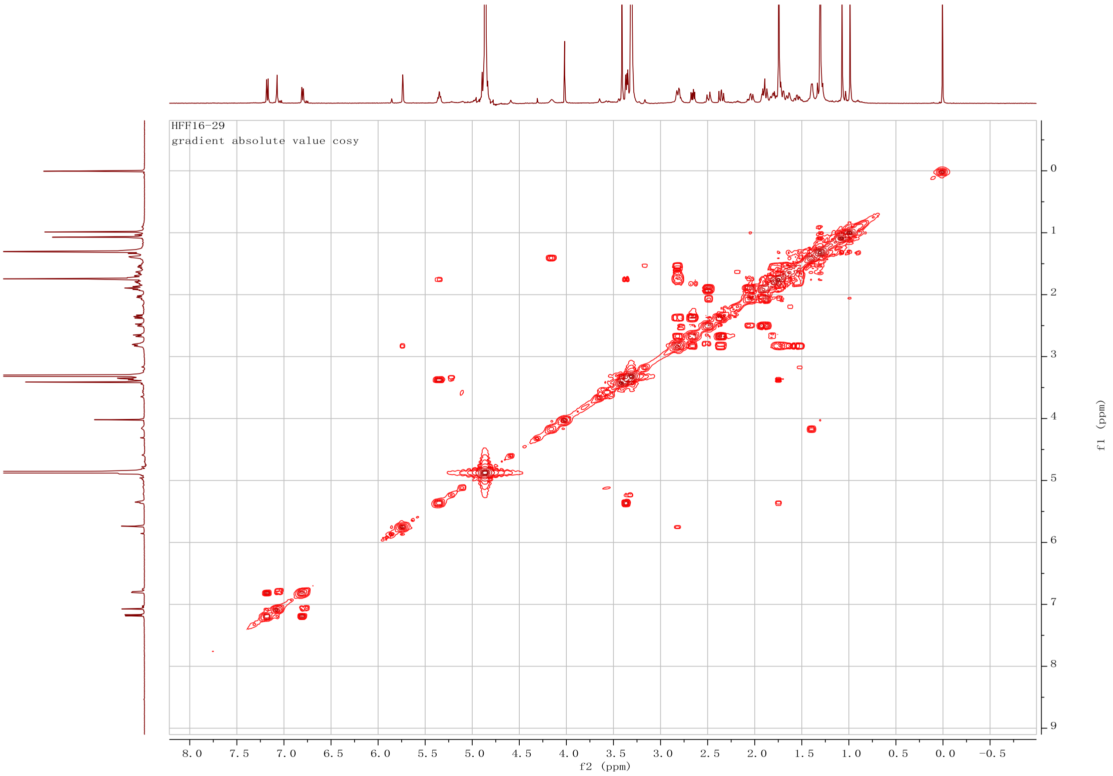

Figure S15. HMBC spectrum of 2 in CD_3OD

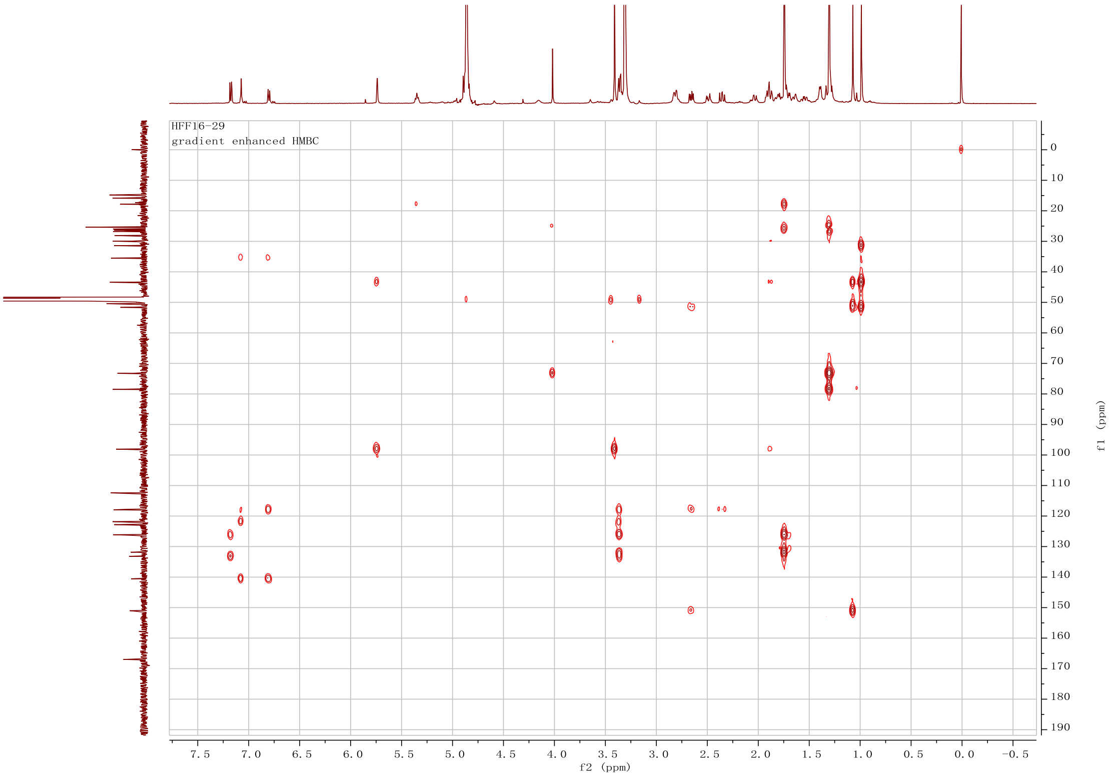

Figure S16. NOESY spectrum of 2 in CD_3OD

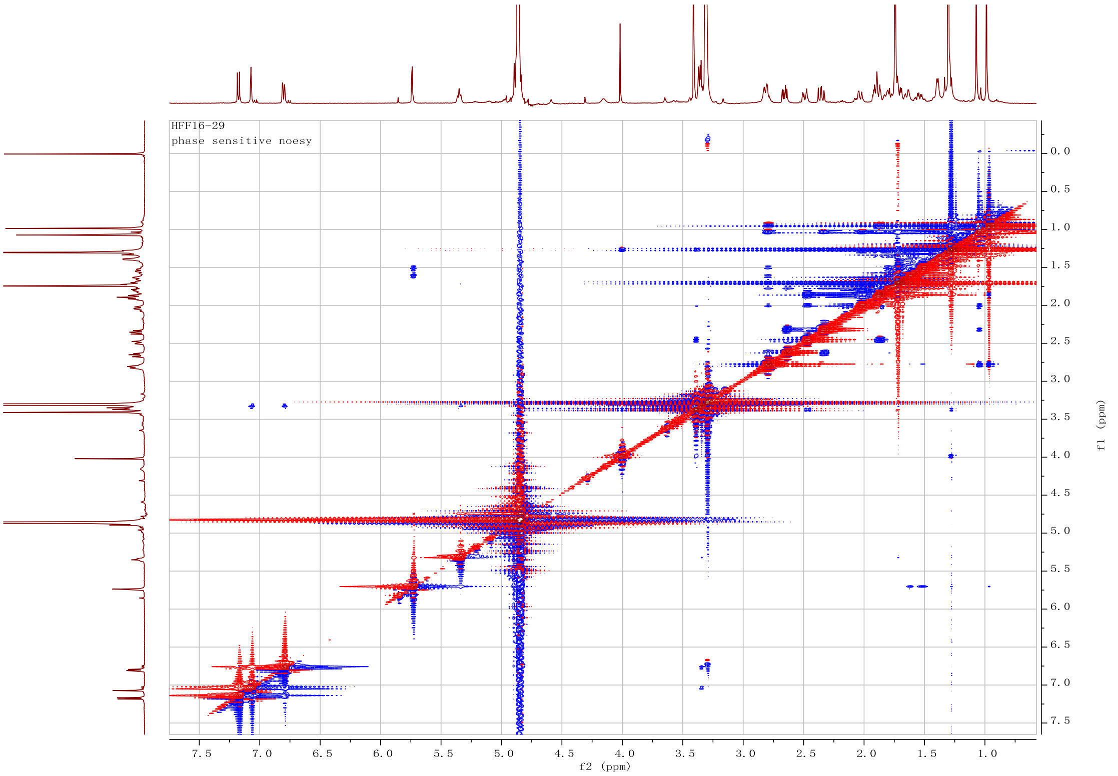

Figure S17. HRESIMS spectrum of 2

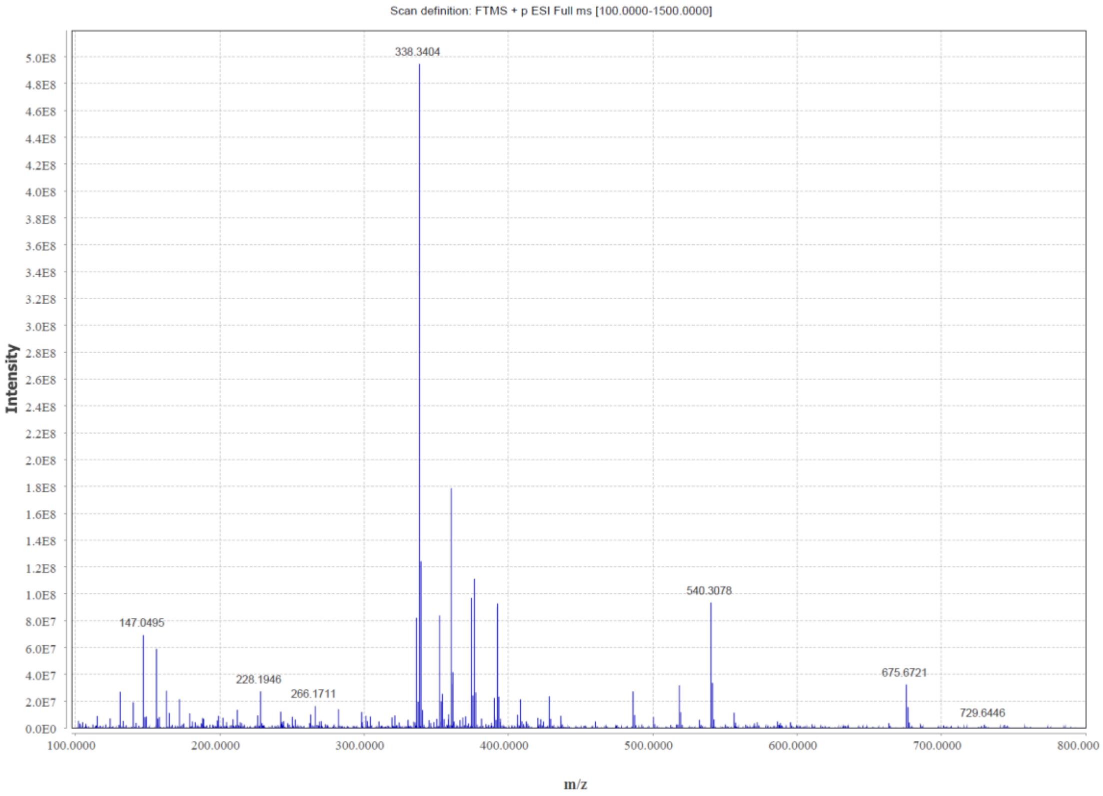

Figure S18. ^{1}H-NMR spectrum of 3 in CD_3OD

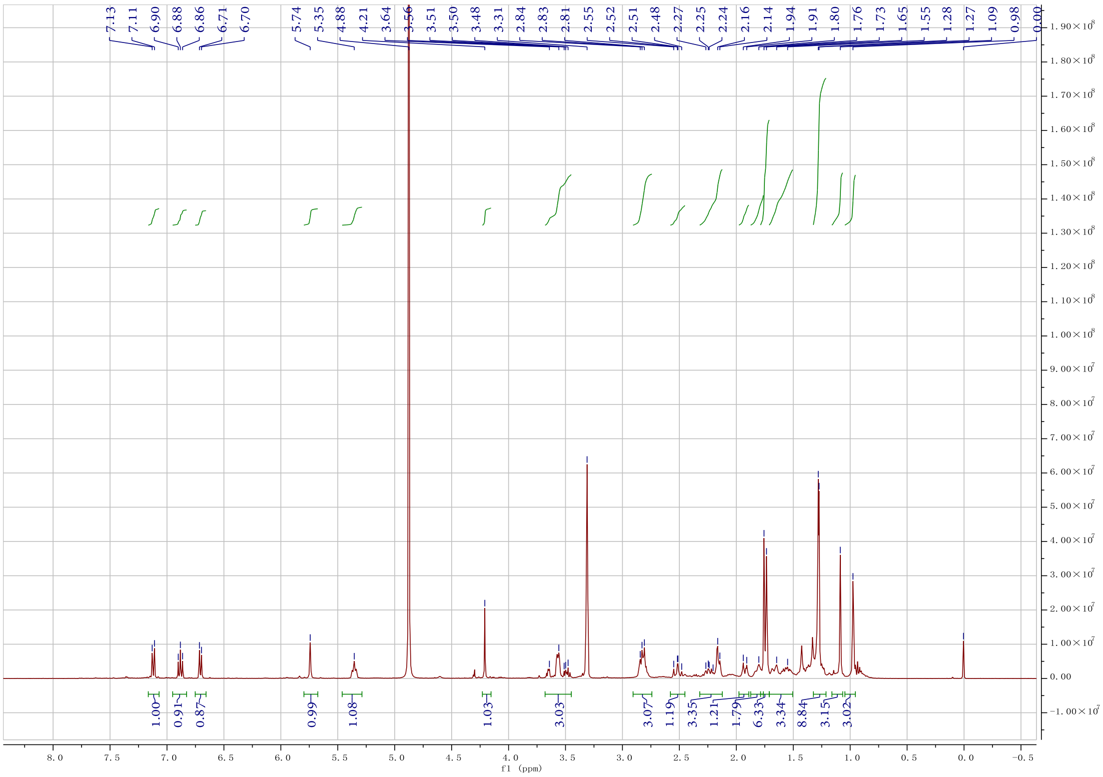

Figure S19. ^{13}C-NMR spectrum of 3 in CD_3OD

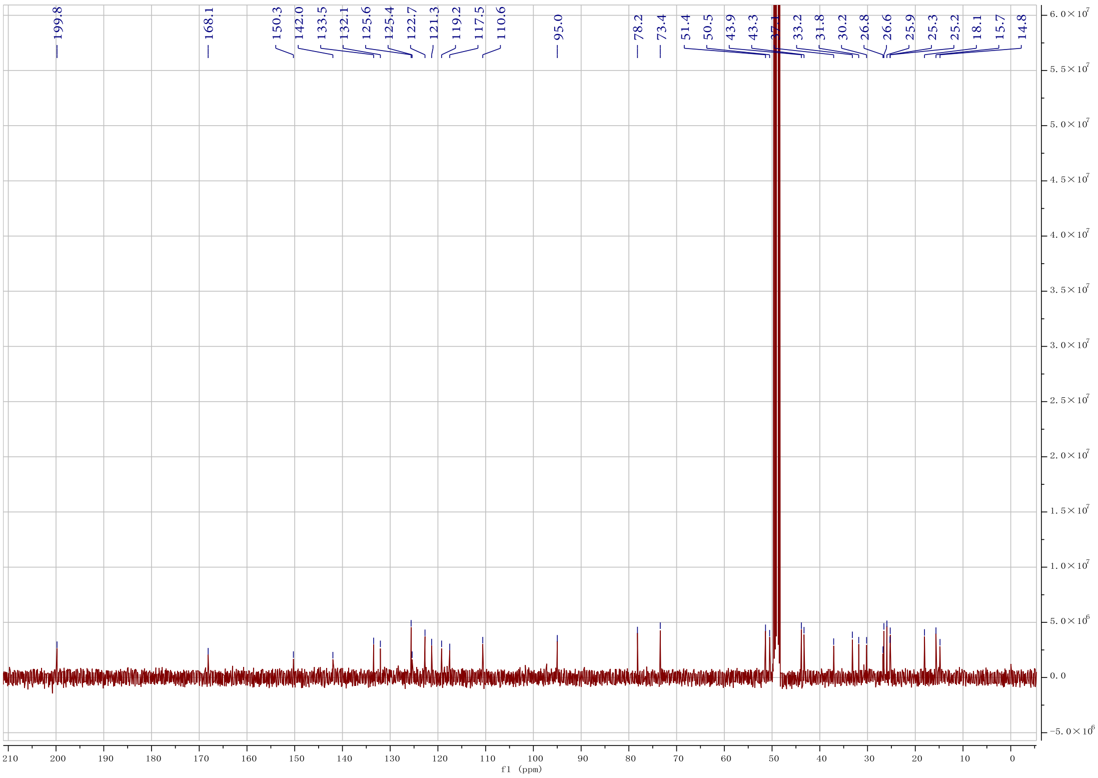

Figure S20. DEPT spectrum of 3 in CD_3OD

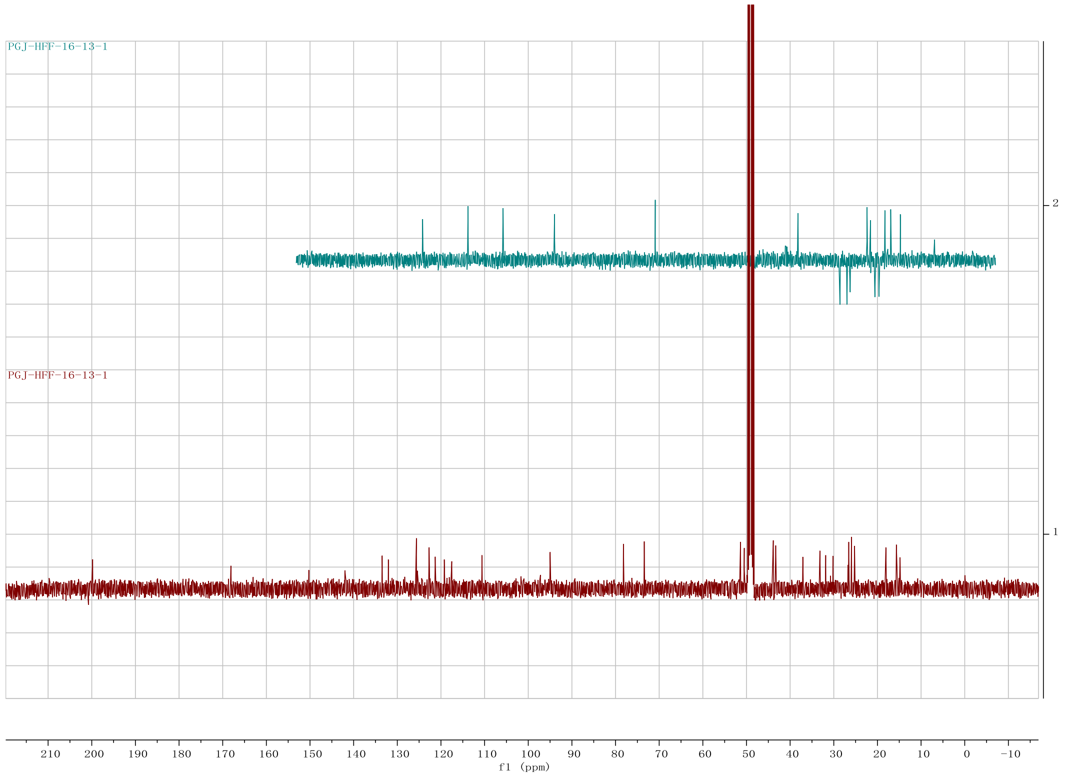

Figure S21. HMQC spectrum of 3 in CD_3OD

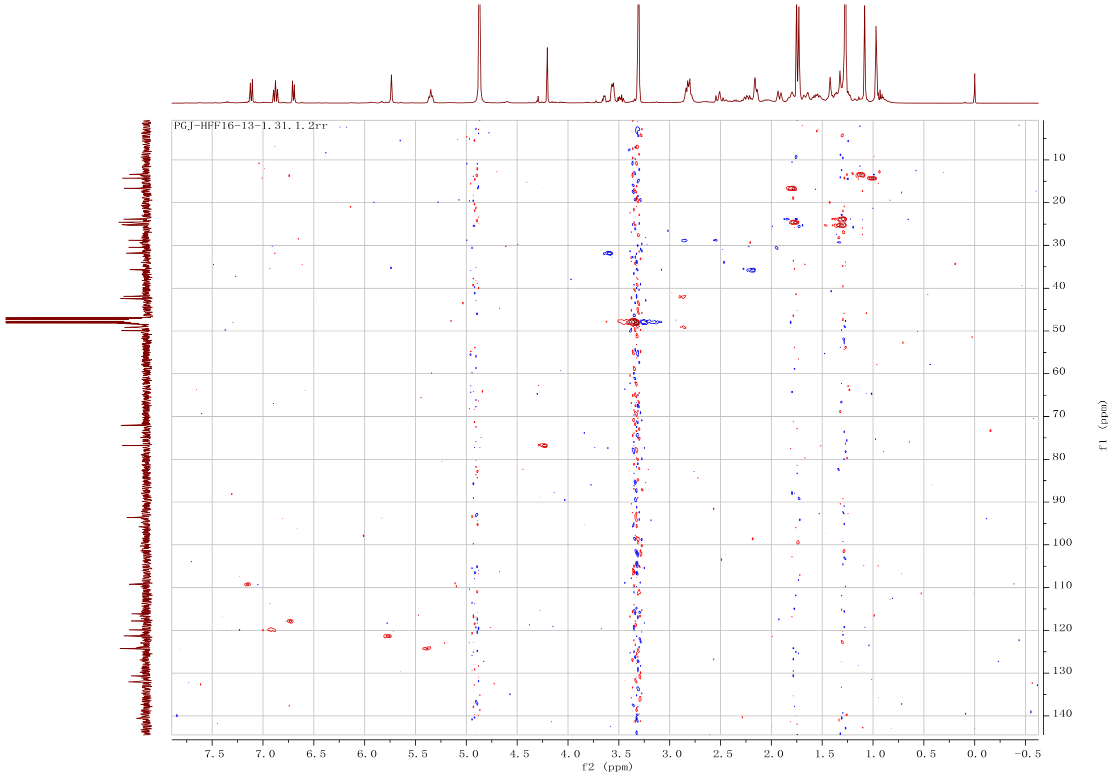

Figure S22. ^{1}H-^{1}H COSY spectrum of 3 in CD_3OD

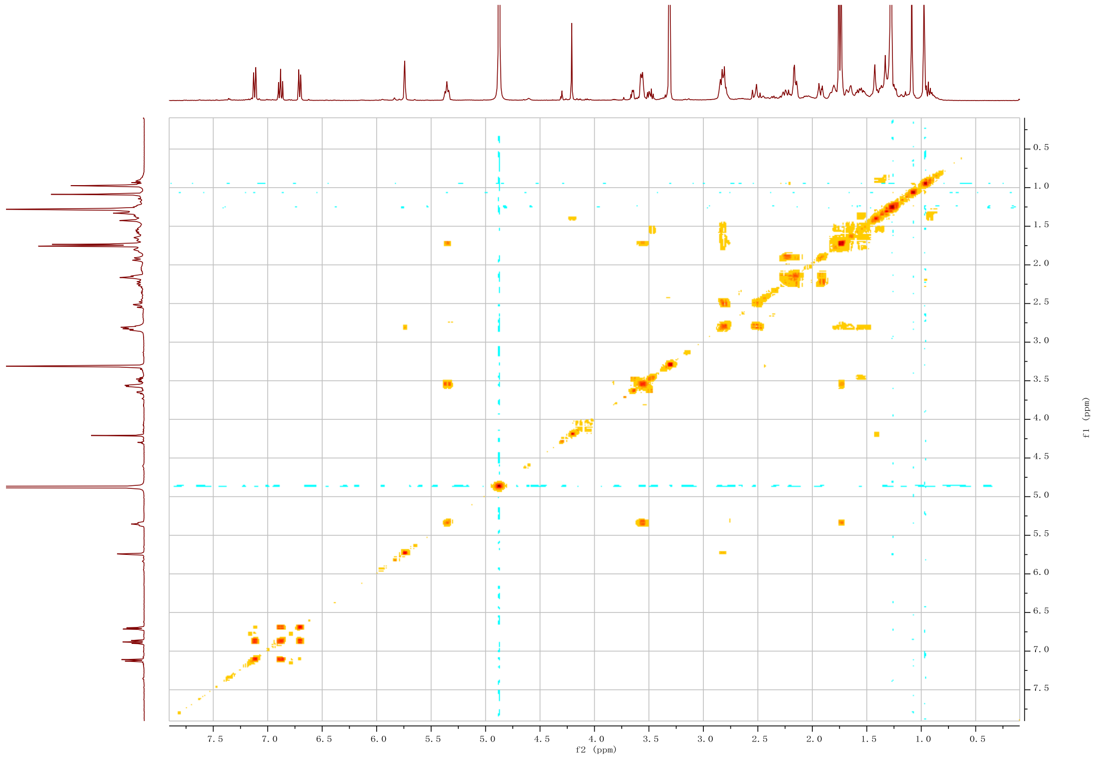

Figure S23. HMBC spectrum of 3 in CD_3OD

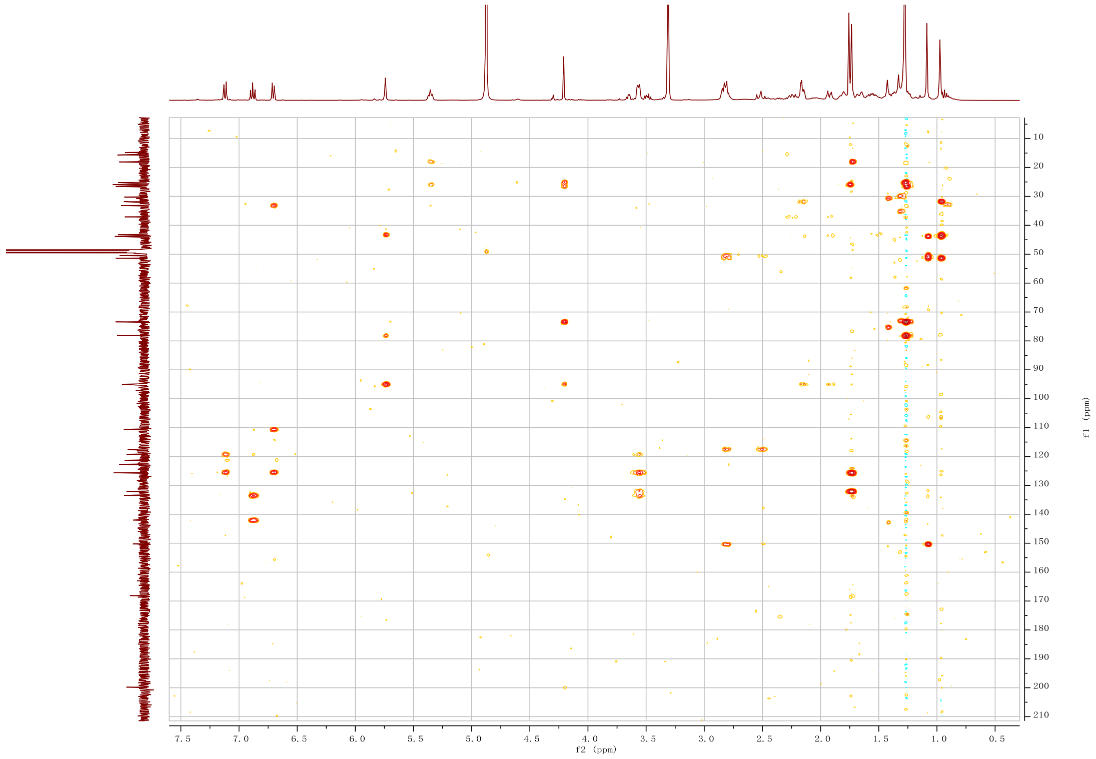

Figure S24. NOESY spectrum of 3 in CD_3OD

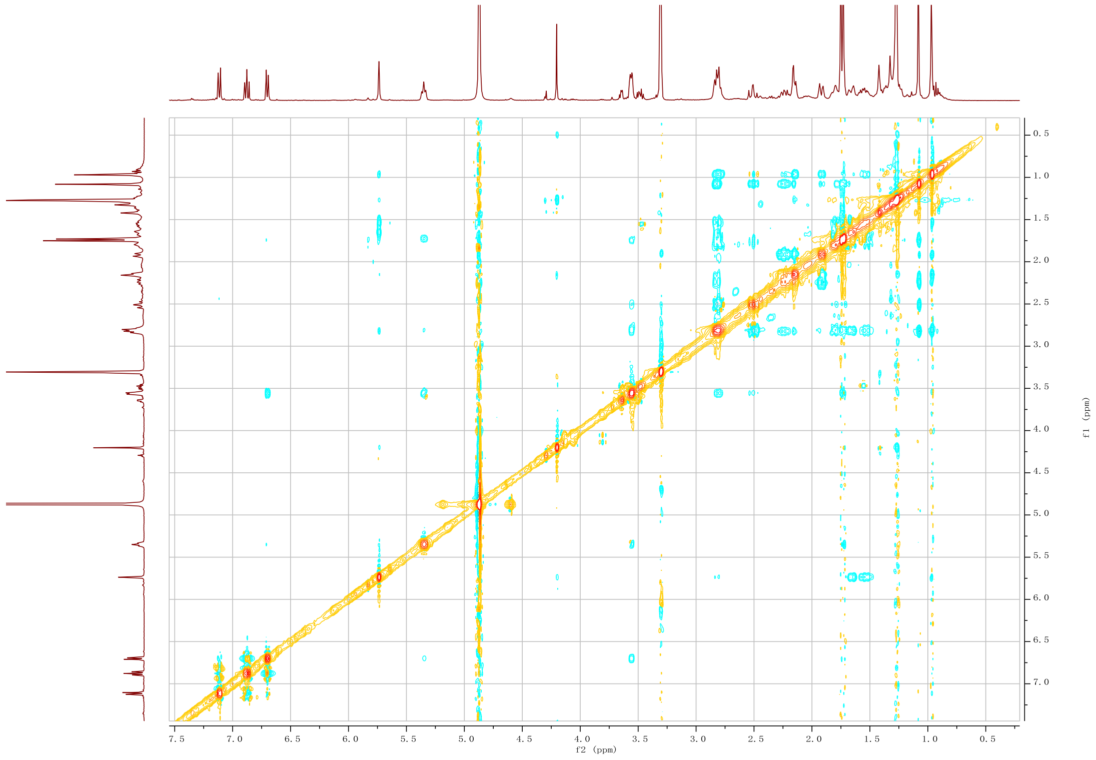

Figure S25. HRESIMS spectrum of 3

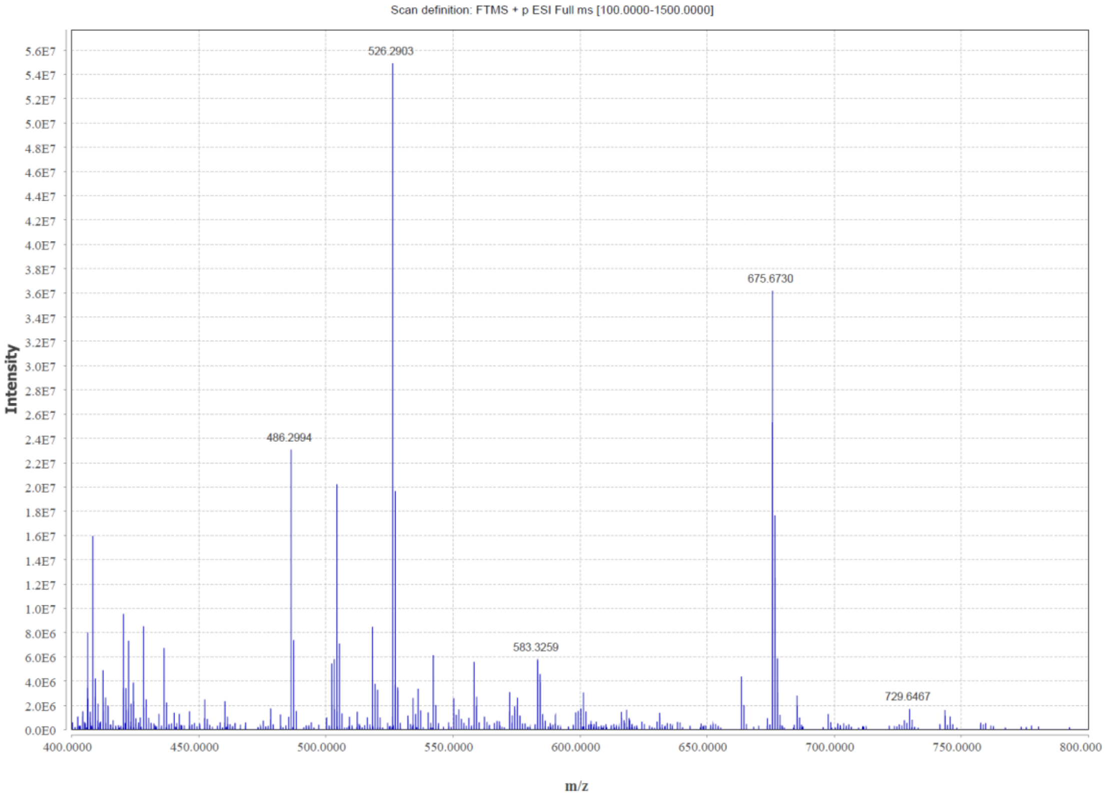

Figure S26. ^{1}H-NMR spectrum of 4 in CD_3OD

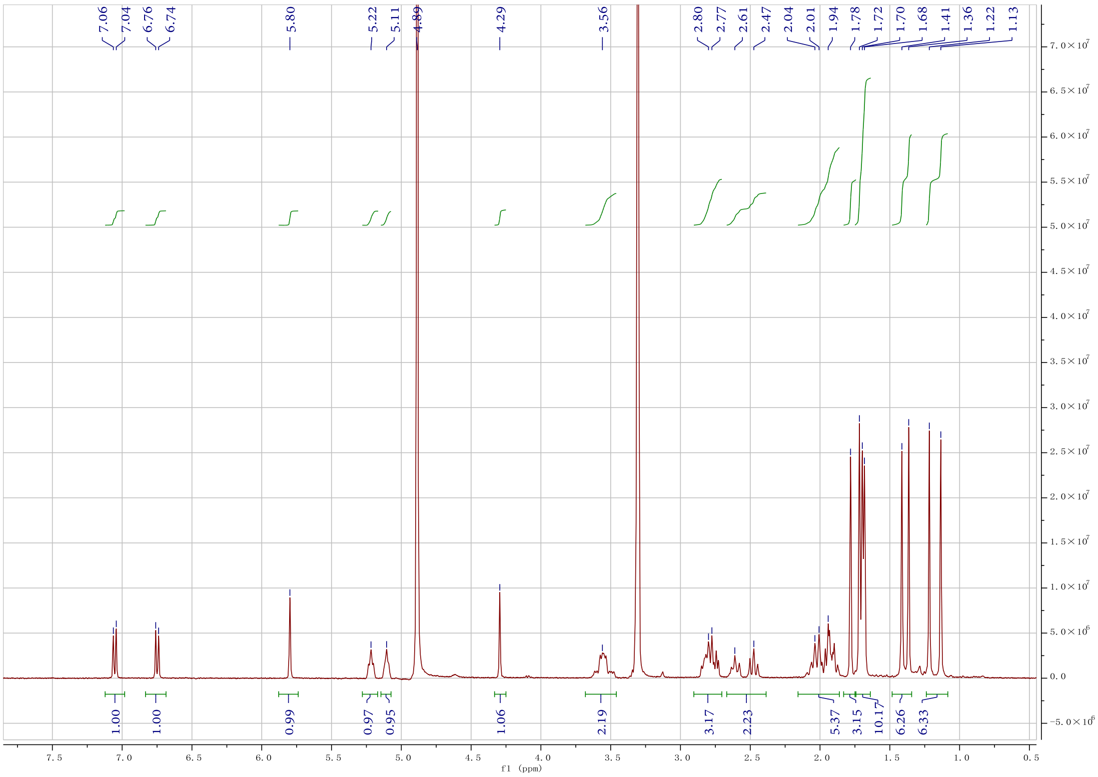

Figure S27. ^{13}C-NMR spectrum of 4 in CD_3OD

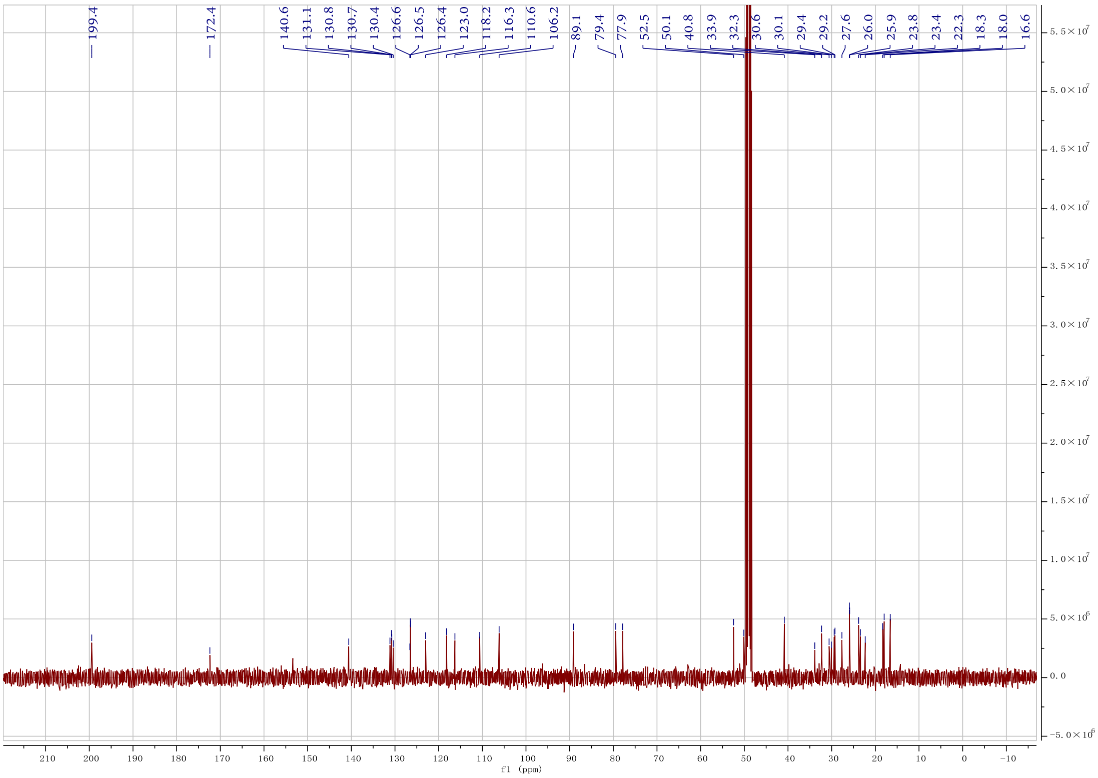

Figure S28. DEPT spectrum of 4 in CD_3OD

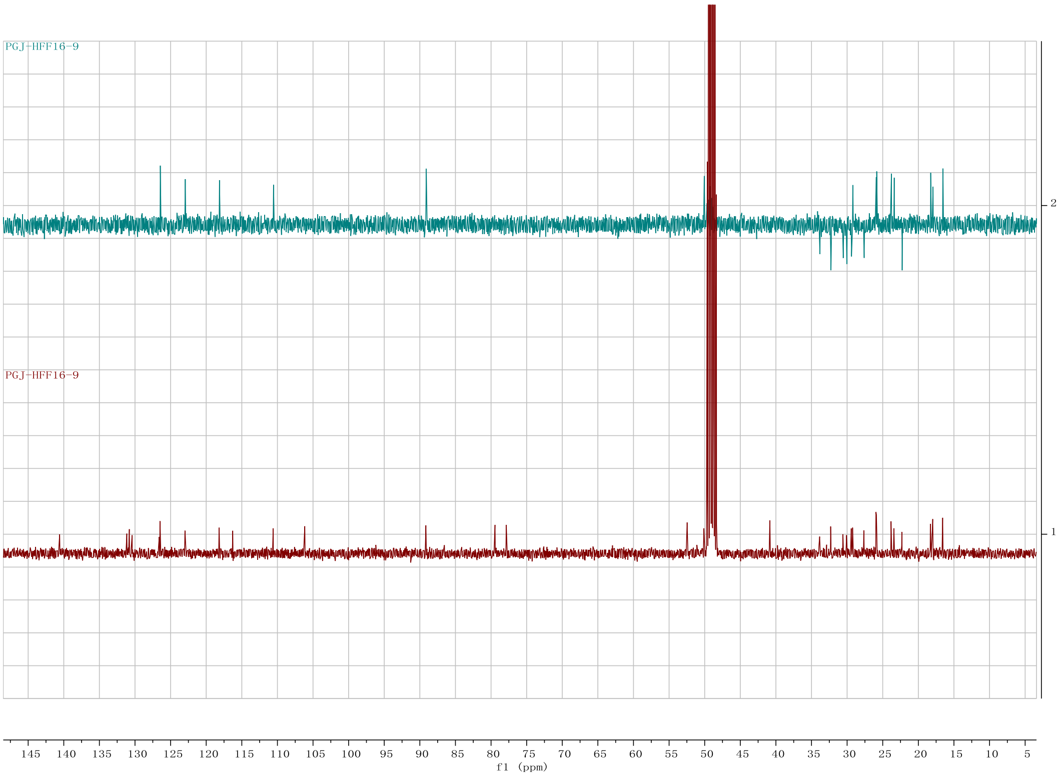

Figure S29. HMQC spectrum of 4 in CD_3OD

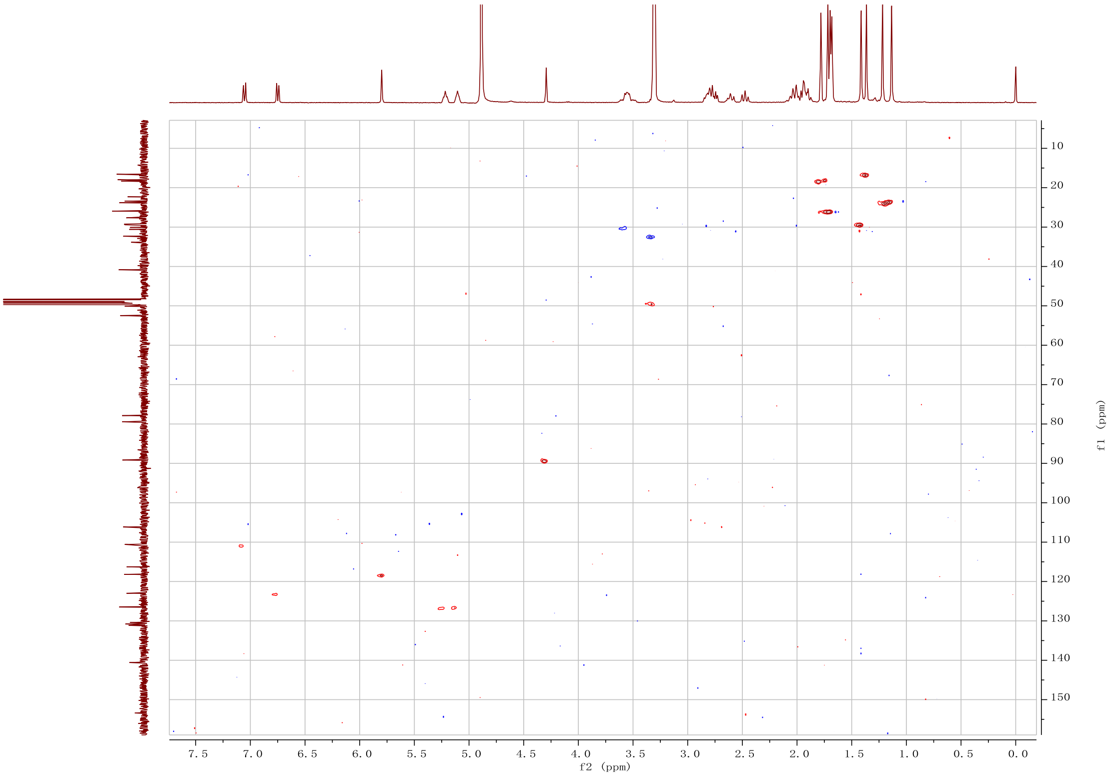

Figure S30. ^{1}H-^{1}H COSY spectrum of 4 in CD_3OD

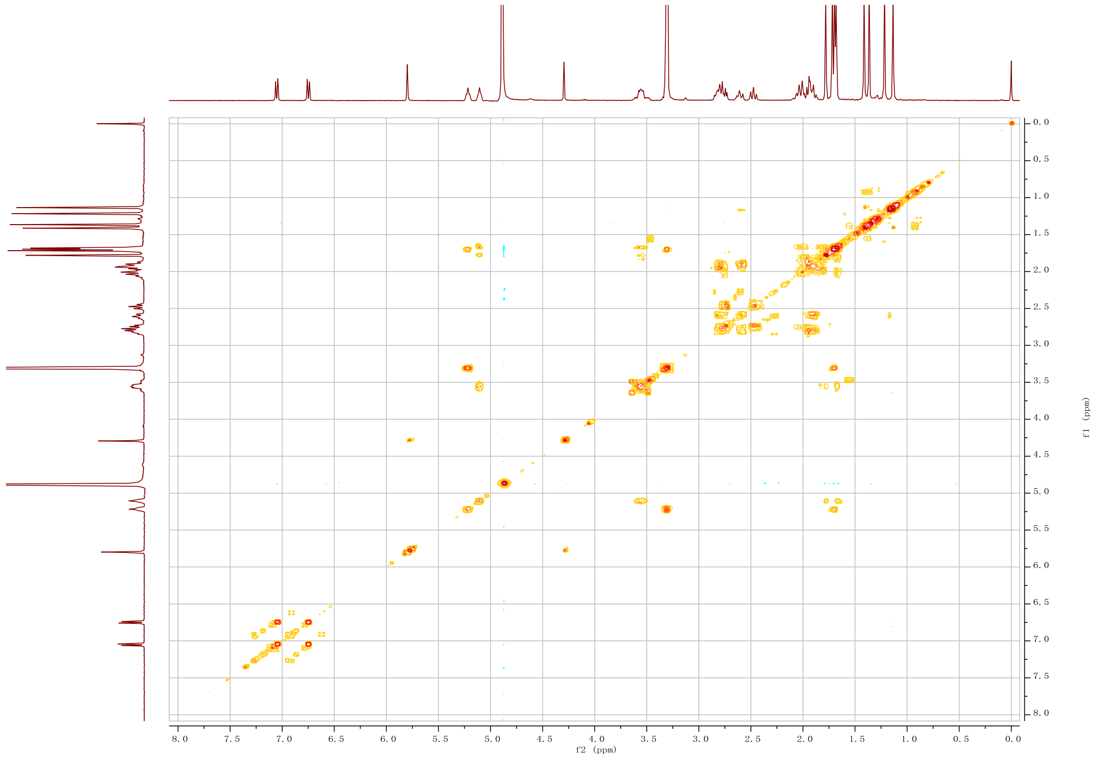

Figure S31. HMBC spectrum of 4 in CD_3OD

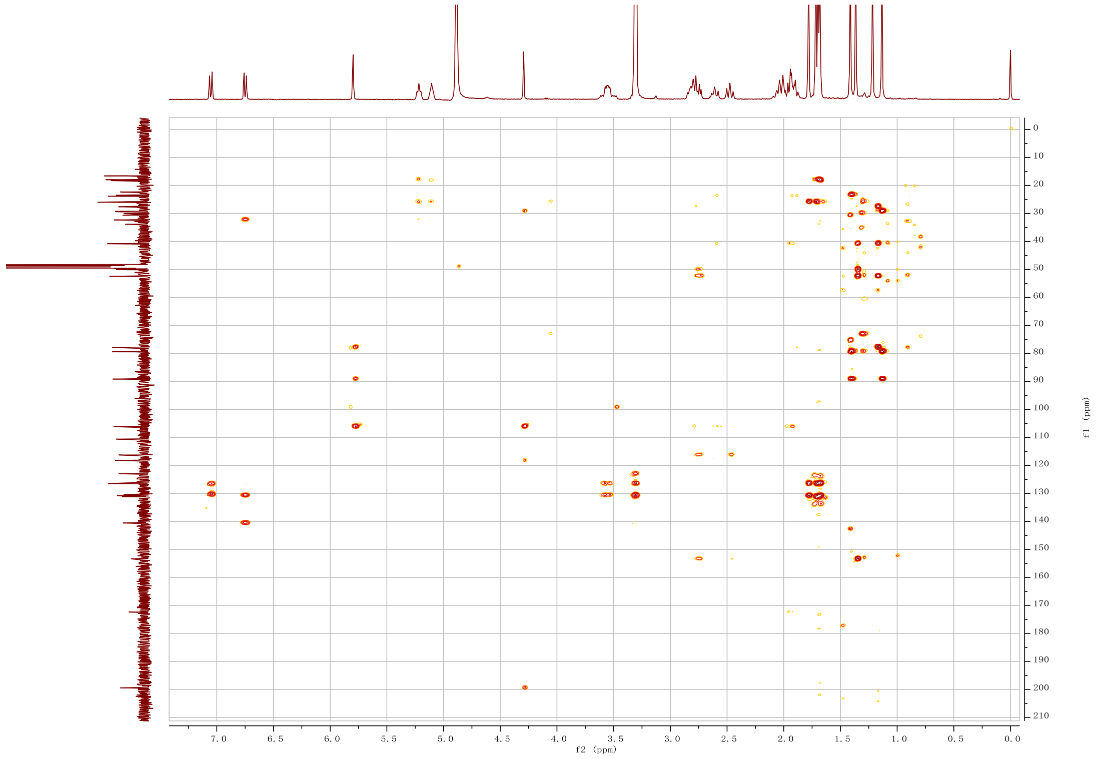

Figure S32. **NOESY spectrum of 4 in CD_3OD**

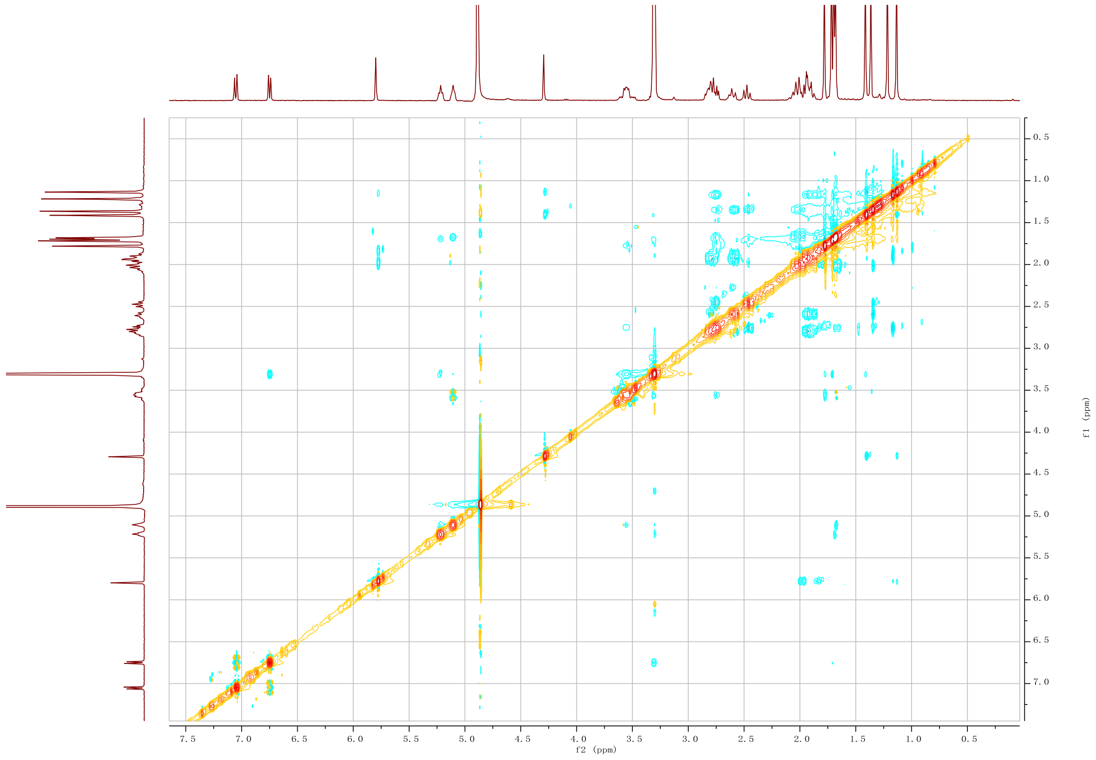

Figure S33. **HRESIMS spectrum of 4**

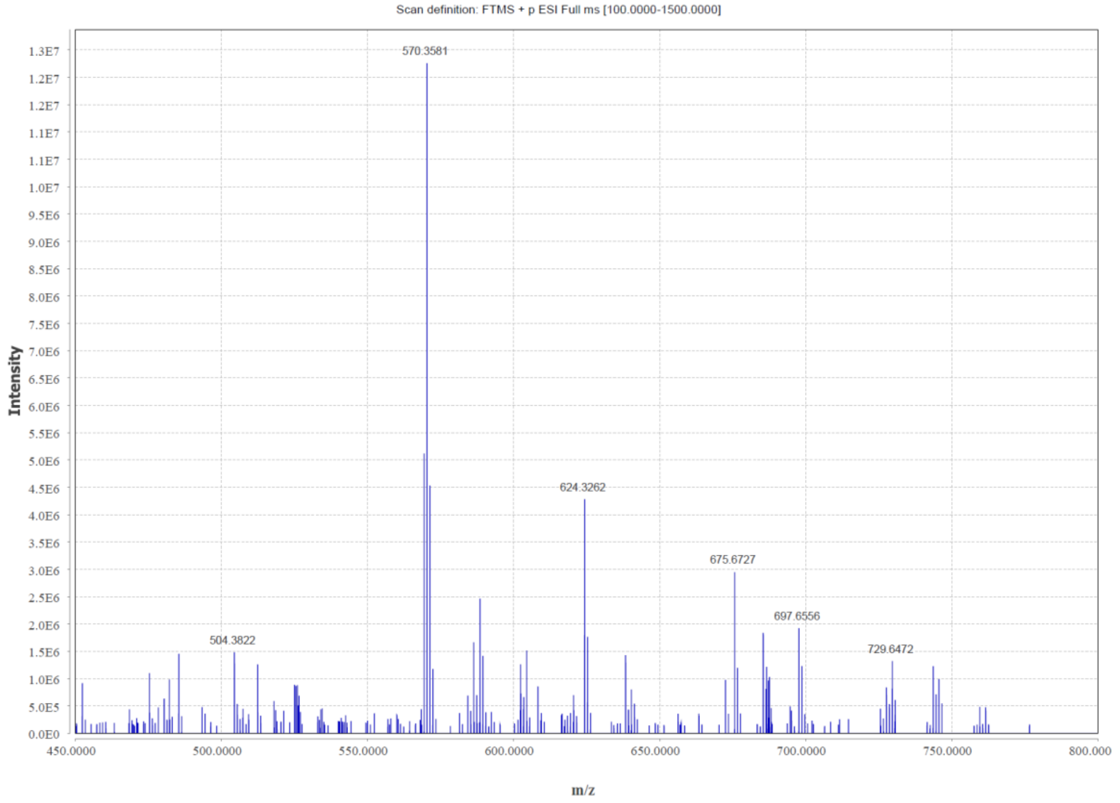

Supplement: Supplementary file 1 [file Data_Sheet_1.PDF]
